# Temporal Changes in Nucleus Morphology, Lamin A/C and Histone Methylation During Nanotopography-Induced Neuronal Differentiation of Stem Cells

**DOI:** 10.3389/fbioe.2018.00069

**Published:** 2018-05-31

**Authors:** Soneela Ankam, Benjamin K. K. Teo, Grace Pohan, Shawn W. L. Ho, Choon K. Lim, Evelyn K. F. Yim

**Affiliations:** ^1^Department of Biomedical Engineering, National University of Singapore, Singapore, Singapore; ^2^Mechanobiology Institute Singapore, National University of Singapore, Singapore, Singapore; ^3^Department of Chemical Engineering, University of Waterloo, Waterloo, ON, Canada; ^4^Department of Surgery, National University of Singapore, Singapore, Singapore

**Keywords:** mesenchymal stem cells, nucleus, embryonic stem cells, mechanotransduction, neuronal differentiation, nuclear changes, epigenetics

## Abstract

Stem cell differentiation can be regulated by biophysical cues such as nanotopography. It involves sensing and integration of these biophysical cues into their transcriptome with a mechanism that is yet to be discovered. In addition to the cytoskeletal and focal adhesion remodeling, nanotopography has also been shown to modulate nucleus morphology. Here, we studied the effect of nanotopography on the temporal changes in nuclei of human embryonic stem cells (hESCs) and human mesenchymal stem cells (hMSCs). Using a high throughput Multi-architecture (MARC) chip analysis, the circularity of the stem cell nuclei changed significantly on different patterns. Human ESCs and MSCs showed different temporal changes in nucleus morphology, lamin A/C expression and histone methylation during topography-induced neuronal differentiation. In hESCs, the expression of nuclear matrix protein, lamin A/C during neuronal differentiation of hESCs on PDMS samples were weakly detected in the first 7 days of differentiation. The histone 3 trimethylation on Lysine 9 (H3K9me3) decreased after differentiation initiated and showed temporal changes in their expression and organization during neuronal differentiation. In hMSCs, the expression of lamin A/C was significantly increased after the first 24 h of cell culture. The quantitative analysis of histone methylation also showed a significant increase in hMSCs histone methylation on 250 nm anisotropic nanogratings within the first 24 h of seeding. This reiterates the importance of cell-substrate sensing within the first 24 h for adult stem cells. The lamin A/C expression and histone methylation shows a correlation of epigenetic changes in early events of differentiation, giving an insight on how extracellular nanotopographical cues are transduced into nuclear biochemical signals. Collectively, these results provide more understanding into the nuclear regulation of the mechanotransduction of nanotopographical cues in stem cell differentiation.

## Introduction

Stem cells *in vivo* reside in a stem cell niche where appropriate biochemical and biophysical cues are present to direct stem cell differentiation (Hsu and Fuchs, 2012). Understanding of how stem cells interact with their extracellular microenvironment will be beneficial for strategies to control stem cell fate (Dalby et al., 2007b; Yim et al., 2007; Teo et al., 2013). Numerous studies using simplified 2D topography models to mimic the native extra-cellular matrix (ECM) have demonstrated that biophysical cues can modulate human embryonic stem cells (hESCs) (Ankam et al., 2013, 2015; Chan et al., 2013a) and human mesenchymal stem cells (hMSCs) (Dalby et al., 2007b; Yim et al., 2007; Engel et al., 2009; Martino et al., 2009; Watari et al., 2012) into different lineages with or without the use of biochemical cues. Other studies have reported the physical continuity from the ECM to the nucleus (Wang et al., 2009; Shivashankar, 2011) and through alteration of the intricate physical network, by mechanical signals, including substrate rigidity, confined cell geometry and topographical perturbations from the ECM, differential gene expression in stem cells can be induced (Engler et al., 2006; Shivashankar, 2011). While studies have provided clues as to how changes in rigidity and cell shape may affect cytoskeletal contractility and nuclear regulation (Engler et al., 2006; Shivashankar, 2011), and how changes in nanotopographical cues may affect cytoskeletal contractility and stem cell differentiation (Teo et al., 2013; Ankam et al., 2015), how stem cells sense and transduce the nanotopographical cues into differential gene remains to be determined. Moreover, the physical continuity between the ECM and the nucleus allows the mechanotransduction mechanism (one form of long range signal transduction within cells) to take place, changing cellular components and collectively producing biochemical signaling pathways, and subsequent cell response to the topographical cues (Maniotis et al., 1997; Crisp et al., 2006; Teo et al., 2013; Ankam et al., 2015).

The plasticity and shape of the nuclei have been shown to correlate with stem cell differentiation; embryonic stem cell nuclei are more plastic than that of fully differentiated cells (Szutorisz and Dillon, 2005). Pajerowski et al. found that after several days in culture, the deformability of ESC nuclei decreased. In fact, the nuclei approached a 6-fold higher relative stiffness in comparison to what is typical of differentiated cells such as embryonic fibroblasts. In addition, the nucleus stiffness was found to be contributed by the nuclear matrix protein, lamin A/C (Pajerowski et al., 2007). This suggested that pluripotent stem cell differentiation was influenced by the change in nucleus mechanical properties, with laminar proteins contributing to the nucleus stiffness (Pajerowski et al., 2007; Heo et al., 2018). A few groups have reported the effects topography has on nuclei shape and gene expression (Dalby et al., 2003, 2007a; Yim et al., 2007). Nuclear lamina also appears to be important in topography-mediated mechanotransduction and consists of a network of lamin proteins and intermediate filaments, similar to cell cytoskeleton (Aebi et al., 1986). In mammals, there are three subtypes of lamin proteins (A-type, B-type and C-type) (Pollard et al., 1990) which could be mechanical linkages that mediate the extracellular topographical cues and stem cells' gene regulatory machinery through direct interaction with DNA-associated proteins or chromatin. (Shoeman and Traub, 1990; Taniura et al., 1995; Zastrow et al., 2004; Dechat et al., 2008) Moreover, previous studies have shown the association of nuclear lamina with the KASH/SUN complex (Shoeman and Traub, 1990; Alberts et al., 1994; Dechat et al., 2008; Tapley and Starr, 2013) which is known to connect to the chromatin based on reviews in Gieni and Hendzel (2008) and Wang et al. (2009). All these studies point out the role of lamin A/C proteins in maintaining differentiated states in stem cells.

Meanwhile, stem cell fate is determined by multi-levels of gene expression patterns involving DNA (Collas, 2010). Nucleosomes containing an octamer of 4 core histone proteins (H2A, H2B, H3, and H4) which are wrapped with DNA base pairs are packed together forming a structure called chromatin. Among these 4 histones, H3 and H4 have been most studied with their combinatorial methylation and acetylation due to their involvement in eukaryotic epigenetic regulatory mechanism (Li, 2002). One type of H3 histone, H3K9 (Lysine9 of histone H3), can exist in mono-, di- or tri-methylation states of which within the chromatin itself, the 3 states are spatially regulated (Bannister and Kouzarides, 2004). The activation or silencing of stem cell genes is controlled by acetylation and/or methylation modifications. For example, in mouse (Melcer et al., 2012) and human (Xie et al., 2013; Qiao et al., 2015) embryonic stem cells, increasing methylation of H3K9 and reducing acetylation of histone can affect chromatin plasticity. Moreover, it has been shown that gene activation has a correlation with the acetylation of H3K9, while gene silencing is associated with the enrichment of H3K9 methylation modification (Melcer et al., 2012; Xie et al., 2013; Qiao et al., 2015), which also influences gene expression and chromatin structure; both H3K9 acetylation and methylation modifications are abundant in MSCs. Furthermore, histone acetylation was shown to increase in hMSCs that were cultured on stretched elastic membranes and microgrooves, while histone deacetylase enzymatic activity was shown to decrease (Li et al., 2011). Understanding epigenetic modifications in relation to topography-induced differentiation and subsequent stem cell fate determination is critical; more studies on epigenetic modifications in specific loci are cited here (Dai and Rasmussen, 2007; Tan et al., 2009; Bannister and Kouzarides, 2011; reviewed in Huang et al., 2015).

Indeed, histone changes in differentiated hMSCs were not isolated to the expression of lamin A/C (Li et al., 2011), and lamin A/C expression as a marker of mouse and human embryonic stem cell differentiation is evident (Constantinescu et al., 2006). From a mechanobiology perspective, hESCs and hMSCs have different differentiation potentials which are reflected by the difference in the nuclear shape, nuclear lamin A/C expression in the nuclear envelope, and histone modification. In this study, we investigate the changes in nucleus morphology and the role of histone modifications within the chromatin, pertaining to topography induced stem cell differentiation and function. We hypothesize that nanotopography will have an effect on stem cell nuclear shape, which will subsequently change gene expression and modulate differentiation; in particular, it is hypothesized that nanotopography will increase the expression of lamin A/C and affect H3K9 methylation in hESCs and hMSCs during nanotopography-induce differentiation. We first utilized a high throughput multi-topography array, a Multi-ARChitecture (MARC) chip consisting of 16 micro- and nano-sized topographical patterns, designed to examine changes in the nuclear shape of stem cells on different types of topographies. We further studied the temporal changes in the lamin A/C expression and H3K9 methylation throughout the topography-induced differentiation of stem cells on 250 nm grating patterns. The understanding of changes in nuclear shape, structure, and H3K9 methylation will allow us to have a better understanding of the temporal events in how topographies affect stem cells during the early stages of differentiation.

## Materials and methods

### Fabrication of multi-architecture (MARC) chip and nanogratings

A customizable multi-architecture chip (MARC) consisting of topographical patterns with different geometries and dimension was designed to examine cell responses to topographies in the same conditions on one chip. The detailed fabrication of MARC chip was previously described (Ankam et al., 2013). In brief, micro- and nano-sized isotropic and anisotropic topographies were imprinted on polycarbonate (PC) via nanoimprint lithography (NIL). In this study, 13 patterns were selected to be examined (Supplementary Figure 1) with technical replicas on each chip. Selected patterns were cut into 2 × 2 mm pieces and assembled into a multi-array on a silicone substrate with two unpatterned fields. Polydimethylsiloxane (PDMS, Sylgard 184, Corning) was used as a binding material. The pattern field area of 2 × 2 mm was selected as it would provide a sufficient cell number on each pattern for statistical analysis, when cells would be at sub-confluence cell density, while a chip consisting of 36–49 patterned areas would still be small enough to fit into a typical 6 well or 12 well-plate. The MARC chip master was surface-treated with perfluorodecyltrichlorosilane (FTDS) and 0.01% Triton X-100 (Sigma Aldrich) prior to using it for fabrication. In order to fabricate a MARC chip replica with the same set of topographies as the imprinted PC fields, a double replication was performed. The mirror image of a MARC chip was replicated with PDMS in a 5:1 ratio of elastomer to crosslinking agent and cured overnight at 60°C. It was then demolded at room temperature. The mirror MARC chip was surface-treated with FTDS and 0.01% Triton X, and used as a master mold for the replication of the MARC chip with PDMS in a 10:1 ratio of elastomer to crosslinking agent. Similar to the master, the sample was cross-linked overnight at 60°C, and demolded at room temperature.

As enhanced topography-induced neuronal differentiation was observed in stem cells cultured on 250 nm gratings in previous studies (Ankam et al., 2013, 2015; Teo et al., 2013, 2014), the temporal analysis of the stem cell differentiation was performed on 250 nm gratings and unpatterned PDMS controls. The 250 nm gratings (250 nm linewidth, gap, and height) PDMS samples were fabricated using soft lithography with a ratio of 10:1 of elastomer to crosslinking agent. The degassed mixture was poured over a nanoimprinted poly methyl methacrylate (PMMA) master mold before being degassed for 1 h. Subsequently, it was cured at 60°C overnight, before demolding at room temperature.

### Verification of the replicated PDMS samples by scanning electron microscope (SEM)

The fidelity of topographical structures replicated using soft lithography was verified using a SEM (JEOL, JSM-6700F). Samples were coated for 20 s using a gold coating machine (JEOL, JFC-1200) to achieve a gold film thickness of approximately 10 nm. The structures were viewed using an accelerating voltage of 5 kV at a working distance of 6 mm.

### Cell culture

#### Human embryonic stem cell maintenance and neuronal differentiation

Human embryonic stem cell line H1 (WiCell Institute, Madison, http://www.wicell.org) was routinely cultured in a mTeSR1 medium (Stem Cell Technologies) on a feeder free system using hESC-qualified matrigel (BD Biosciences) (Hughes et al., 1886). The medium was changed once daily. After manual removal of differentiated cell colonies using an inverted microscope (Nikon TS100) and a flame-bent Pasteur pipette, colonies passaging was done with dispase (1 mg/ml). The cells were passaged at a ratio of 1:4–1:6. The pluripotency of the hESCs were characterized routinely before the differentiation experiment (Supplementary Figure 3) with the list of antibodies provided in Supplementary Table 1. Removal of the differentiated colonies was done carefully before cell seeding was done onto the MARC chip or grating samples in order to ensure uniform starting population of undifferentiated cells and to avoid biased outcomes.

Prior to cell seeding, air plasma treatment (Harrick expanded plasma cleaner) was done on PDMS samples for 30 s at 10.5 W. Following that, samples were sterilized by UV irradiation, and were incubated overnight with 33 μg/ml poly-L-ornithine (PLO, Sigma) at 37°C and with 20 μg/ml laminin from Engelbreth-Holm-Swarm murine sarcoma (Invitrogen) for 8 h at 37°C (Drago et al., 1991).

The hESCs were then seeded on poly-L-ornithine (PLO) and laminin-coated MARC or patterned and unpatterned PDMS and was grown in N2B27 medium [DMEM/F12 (Bio-Rev Pte. Ltd): Neurobasal medium (Invitrogen) in the ratio 1:1 supplemented with 1X N2 and B27 supplements (Invitrogen)]. Unpatterned PDMS was used as the control. It was estimated that about 10–15 clumps (2 × 10^4^-2.5 × 10^4^ cells) were needed to be seeded per cm^2^ of PDMS substrate. Afterwards, cells were fixed and immunofluorescence stained. The changes in the pluripotency marker (Nanog) and neuronal marker expression were characterized (Supplementary Figure 4). For morphometric analysis on MARC chip, H1 cells were seeded on the MARC chip in single cells in mTeSR1 medium with ROCK inhibitor to promote attachment and survival. The media was changed to DMEM/F12 with KOSR to reduce biochemical signaling.

The H1 hESCs were cultured and differentiated on the samples for 7 days. Temporal analysis for nuclear morphology, H3K9 methylation, and lamin A/C expression were performed on Day 0, 1, 4 and 7. However, for MARC chip analysis, to avoid cell overcrowding on the 2 × 2 mm field of MARC chip, the samples were fixed on Day 5 for image analysis for the nuclear morphology for hESCs on different patterns. As the nuclear area and elongation of hESCs on both tissue culture polystyrene (TCPS) and PDMS substrates were similar (Supplementary Figure 5), and since Day 0 referred to cells before topographical exposure, Day 0 cellular data was obtained from cell culture on TCPS substrate.

#### Human bone marrow mesenchymal stem cells (MSCs)

Bone marrow MSCs (CD166+, CD105+, CD44+, CD29+, CD45–, CD34–, CD14–, Lonza, Poietics) were cultured and expanded in serum containing Mesenchymal Stem Cell Growth Medium (MSCGM, Lonza) according to the Lonza's protocol. The passage number of hMSCs used in all experiments was passage number 4–6. The PDMS samples were air plasma-treated, sterilized and coated with 5 μg/cm^2^ fibronectin (Biological Industries) for 1 h at 37°C prior to cell seeding at 5,000 cells/cm^2^.

The media for hMSC neuronal induction was supplemented with 30 μM retinoic acid (RA, Sigma) from a 40 mg/ml RA stock solution dissolved in dimethylsulfoxide.

### Immunofluorescence analysis of lamin A/C and H3K9 methylation in hESCs and hMSCs on nanotopography

Immunostaining samples were fixed and investigated on Day 0, 1, 4, and 7. Fixing was done using 4% paraformaldehyde in phosphate buffered saline (pH 7.4, PBS) for 15 min and permeabilization was done using 0.1% Triton X-100 for 30 min. To prevent non-specific binding, the samples were subsequently blocked using 10% goat serum and 1% bovine serum albumin (BSA) for 1 h at room temperature prior to incubation with primary antibodies. For hMSCs, mouse anti-lamin A/C antibody (1:100, Abcam) or rabbit anti-histone H3 mono methyl K9 (anti-H3K9me1, 1:500, Abcam) were used; these samples were incubated overnight. Then for hESCs, samples were incubated with mouse anti-lamin A/C (1:100), rabbit anti-histone H3 trimethyl K9 (H3K9me3, 1:200, Abcam), or mouse anti- histone 3 acetylation on Lysine 9 (H3K9ac, 1:500, Abcam). Samples were then incubated with the fluorescent Alexa Fluor 488 goat anti-mouse, or goat anti-rabbit secondary antibodies (1:500, Molecular Probes) at room temperature for 1 h. Counter-staining of nucleus and F-actin was performed using DAPI (1:1,000, Sigma-Aldrich) and Alexa Fluor 546 Phalloidin (1:500, Molecular Probes) respectively for 20 min. Samples were mounted and visualized using Olympus Fluoview FV1000 confocal microscope equipped with 20 mW solid state 405 nm, 25 mW Argon ion 458 nm, 488 nm, 515 nm, and 1 mW HeNe Green 543 nm lasers, or Leica TCS SP5 and Zeiss LSM710 confocal microscope.

### Image analysis

On each of the 250 nm gratings and unpatterned PDMS samples, representative images were captured at four random locations. On MARC chip, tiles imaging was done for the entire field. The calculation of nuclei alignment was done by measuring the angle between the grating axis and the long axis of the nucleus; when the measured angle was below 15°, nuclei were considered to be aligned. Nucleus circularity was calculated with the following formula: Circularity = 4π × area/(perimeter)^2^ by ImageJ software. An elongated nucleus shape will give circularity value close to 0, while a perfect circle will give circularity value of 1. Nucleus elongation was analyzed by calculating the elongation factor *E* = (long axis/short axis) −1, which describes the extent of the equimomental eclipse lengthening of the nucleus due to the nanogratings (Andersson et al., 2003). Imaris was used to determine the nucleus volume as previously described (Makhija et al., 2016). The data were normalized to the nucleus volume of the hESCs on cover-slip for comparison.

For intensity measurements, all images were acquired using predetermined confocal microscopy parameters. Fluorescence images were captured in 6 different planes along the z-axis. Prior to performing Mean Gray Value (MGV) measurement to determine H3K9me1 and lamin A/C fluorescence intensity, the stacks were combined using ImageJ and the maximum pixel gray value intensity was shown on the flattened images. The region of interest was created around the nucleus, and ImageJ module was used for the MGV measurements. The total MGV of a particular pattern was then divided over the number of cells on the particular pattern and was subsequently called Average Mean Gray Value (AMGV). Data were obtained from 3 separate experiments with triplicates for all hMSC experiments on MARC chip and the temporal analysis, except for the H3K9me1 expression in hMSCs on MARC chip where 4 separate experiments were measured.

### Flow cytometry analysis of H3K9me1 and lamin A/C in hMSCs on nanotopography

To ensure the consistency of flow cytometry measurement, a time point T was set to be the time the measurement was taken. Thereafter, hMSCs were seeded at time point T-7, T-4, T-1, and T which corresponds to Day 7, Day 4, Day 1, and Day 0 data respectively. Flow samples were prepared according to the following procedures: At time T, cells were passaged by using trypsin-EDTA (0.25%). A wash buffer (0.1% sodium azide and 0.5% BSA in PBS) was then added to re-suspend the detached cells. Cells were then fixed with 1% PFA on ice for 15 min. After which, samples were washed, vortexed and centrifuged for 5 min at 1,000 rpm and supernatant is then removed. Permeabilization was done using 0.1% Triton-X in PBS on ice for 15 min. Subsequently, samples were blocked with 2% goat serum in PBS on ice for 15 min. The samples were then incubated with primary antibodies against lamin A/C (1:100) or H3K9me1 (1:500) at 4°C for 30 mins and then in secondary antibodies (1:750) with two times washing in between incubation steps to minimize background noise and unspecific binding. Finally, the samples were filtered through a 60 μm nylon filter prior to flow analysis using the DAKO Flow Cytometry Analyzer (Dako Cytomation Cyan LX). Human MSCs cultured on unpatterned PDMS substrates were used as negative controls. The experiments were done three times with at least triplicates in each of the experiment. Representative flow cytometric fluorescence histograms are shown in **Figures 5**, **8**, and Supplementary Figures 6, 7. Mean fluorescence intensity was measured from the fluorescence histograms as previously described (Chan et al., 2013b).

### Statistical analysis

Data were analyzed using one-way ANOVA and Tukey's *post-hoc* analysis. In general, ^*^ represents statistical significance with *p* ≤ 0.05, ^**^ *p* ≤ 0.01, and ^***^ *p* ≤ 0.001. Otherwise, *p*-values will be stated in the figure legend. Sample size (N) will also be reported in the figure legend. Data are shown as average ± standard error mean (SEM) or average ± standard derivation (SD) as indicated in the figure legend.

## Results

### Characterization of MARC chip and 250 nm gratings

The replicated nanotopography was verified using scanning electron microscopy (SEM). Representative SEM images of the MARC chip are shown in Supplementary Figure 1. The temporal analyses of the changes in the stem cell nuclei were performed on 250 nm gratings and unpatterned PDMS controls. The PDMS nanogratings fabricated by soft lithography showed good pattern fidelity (**Figure 2A**).

### Nucleus morphology on MARC chip

**Figure 1:**
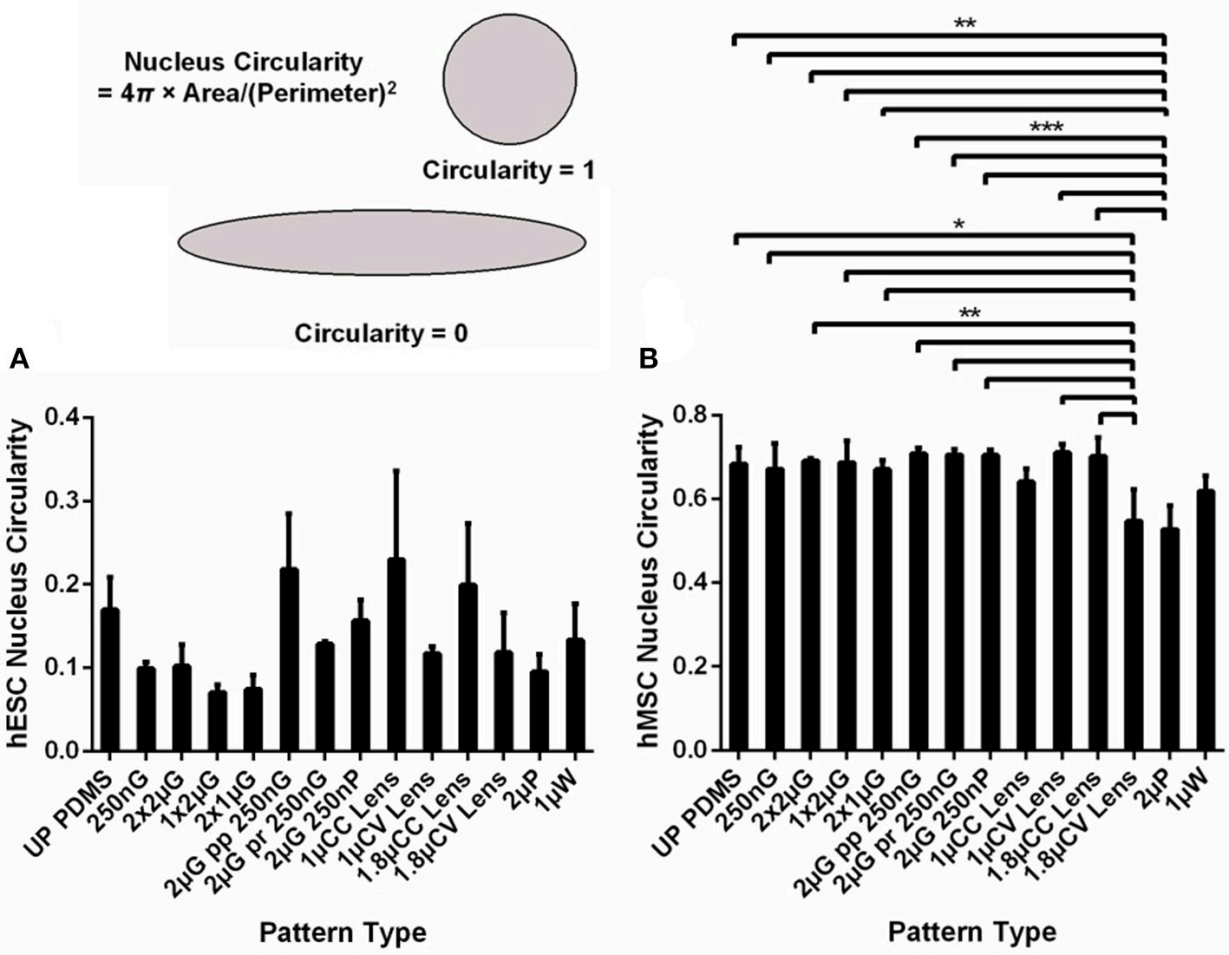
The nucleus circularity index, with values closer to 1 indicate a higher circularity, of **(A)** human embryonic stem cells (hESCs) and **(B)** human mesenchymal stem cells (hMSCs) cultured on the MARC chip for 5 and 7 days, respectively. All data shown as average ± SD. Comparison of experimental replicas **(A)**, *N* = 2 and **(B)**, *N* = 3 with at least 100 cells analyzed in each of the experimental replica on each pattern type. ^*^Represents statistical significance with *p* ≤ 0.05, ^**^p ≤ 0.01, ^***^ *p* ≤ 0.001.There was no statistical significant difference found between hESCs circularity on different pattern types. Refer to Table 1 for abbreviations.

The changes in the cytoskeleton could be correlated with the changes in the nucleus. The circularity index of nuclei of the stem cells on various topographical patterns on the MARC chip was measured with values closer to one indicating a higher circularity. After 5 days of culture on the MARC chip with minimal biochemical signals, the shape of the hESCs nuclei appeared to change on different patterns (Figure 1A). Differences in the nucleus circularity were observed among the hESCs on different patterns. For example, the anisotropic gratings decrease the circularity of the nuclei. The nuclei of the hESCs on hierarchical patterns and isotropic patterns, such as wells, and the unpatterned control exhibited a higher circularity index as compared to gratings topography. Interestingly, hESCs on the concave microlenses, either in 1 or 1.8 μm diameter, showed a higher nuclear circularity compared to the hESCs on the convex microlenses with the same dimension.

**Table 1:** List of MARC chip topographies and their abbreviations.

**MARC chip topography**	**Abbreviations**
250 nm lines, 250 nm space, 250 nm depth	250 nG
2 μm lines, 2 μm space, 2 μm height	2 × 2μG
1 μm lines, 2 μm space, 120 nm height	1 × 2μG
2 μm lines, 1 μm space, 80 nm height	2 × 1 μG
2 μm line ⊥ 250 nm line	2 μG pp 250 nG
2 μm line//250 nm line	2 μG pr 250 nG
2 μm lines 250 nm pillar	2 μG 250 nP
1 μm pitch 0.3 μm sag microlens concave	1 μCC Lens
1 μm pitch 0.3 μm sag microlens convex	1 μCV Lens
1.8 μm diameter, 2 μm pitch, 0.7 μm sag microlens concave	1.8 μCC Lens
1.8 μm diameter, 2 μm pitch, 0.7 μm sag microlens convex	1.8 μCV Lens
2 μm pillars, 12 μm pitch, 2 μm height	2 μP
1 μm holes, 6.5 μm pitch, 1 μm depth	1 μW
Unpatterned control	UP PDMS

The circularity of the hMSCs nuclei was analyzed after 7 days of culture on MARC chip in MSC growth medium (Figure 1B). In contrast to hESCs, the nuclei of hMSCs on different patterns showed a smaller range of variation. A significant decrease was observed in hMSCs cultured on 1 μm concave and 1.8 μm convex lenses (*p* ≤ 0.05, *N* = 3) and 2 μm pillars (*p* ≤ 0.01, *N* = 3). The circularity index values were around 0.7 on both anisotropic patterns and unpatterned control. Representative immunofluorescence images of the nuclei on selected topographies are shown in Supplementary Figure 2.

### Temporal changes in the nucleus morphology and size during neuronal differentiation of hESCs

**Figure 2:**
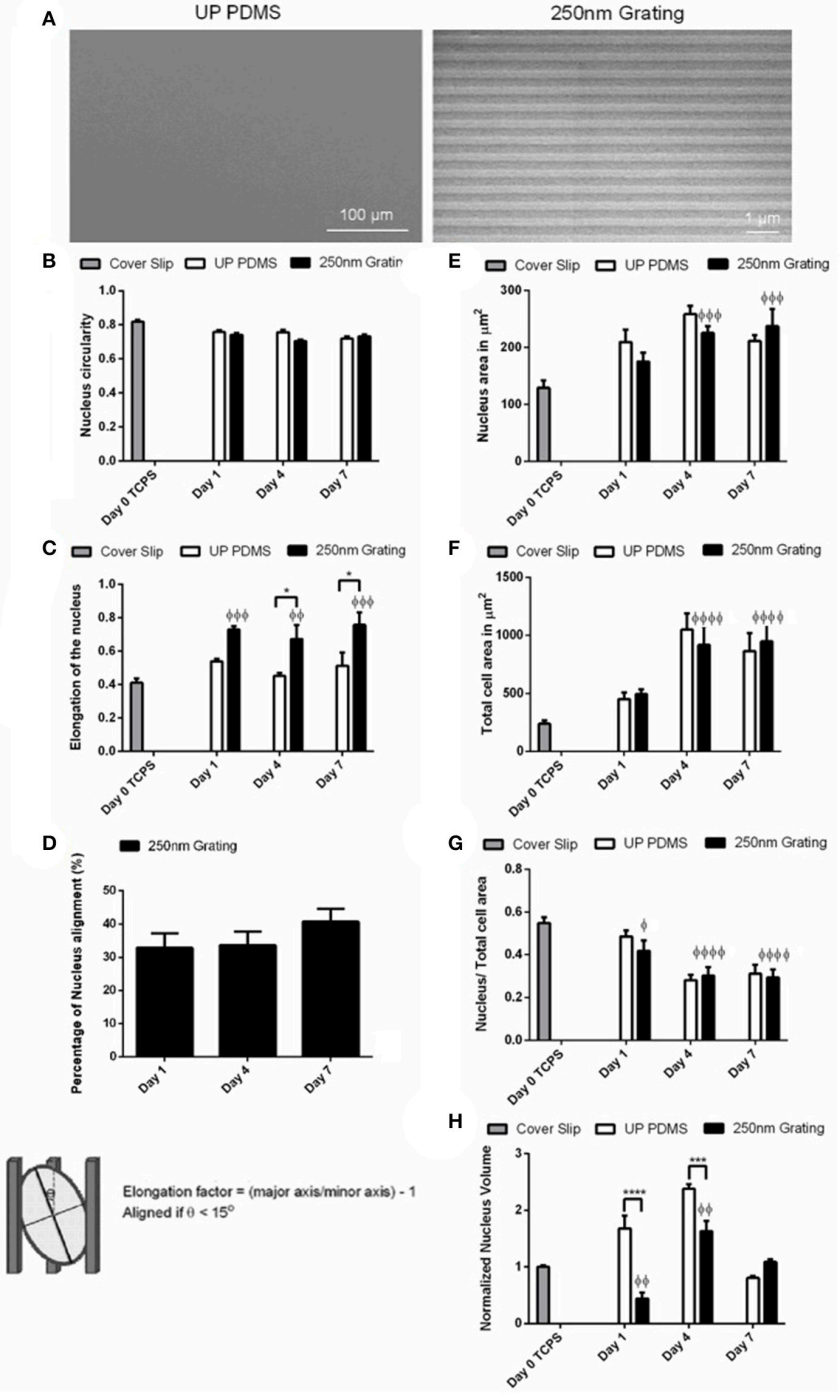
**(A)** Scanning electron microscopy (SEM) image of unpatterned PDMS substrates (control) and 250 nm gratings. Temporal changes of the **(B)** nucleus circularity (comparison of replica mean values, *N* = 3), **(C)** nucleus elongation (comparison of replica mean values, *N* = 4), **(D)** nucleus alignment (comparison of experimental replicas, *N* = 3), **(E)** area of nucleus (comparison of experimental replicas, *N* = 3), **(F)** total cell area (comparison of experimental replicas, *N* = 3), **(G)** the ratio of nucleus area to total cell area (comparison of experimental replicas, *N* = 3), **(H)** normalized nucleus volume against Cover Slip control (comparison of experimental replicas, *N* = 4) were measured on hESCs grown on unpatterned and nano-patterned substrates during differentiation. At least 50 cells were analyzed in each of the experimental replica. ^*^Represents statistical significance with *p* ≤ 0.05 against unpatterned PDMS, ^***^Represents statistical significance with *p* ≤ 0.001 against unpatterned PDMS, ^****^p ≤ 0.0001 against unpatterned PDMS, ^φ^*p* ≤ 0.05 against Cover Slip control, ^φφ^*p* ≤ 0.01 against Cover Slip control, ^φφφ^*p* ≤ 0.001 against Cover Slip control, and ^φφφφ^*p* ≤ 0.0001 against Cover Slip control. Data are shown as average ± SEM.

To understand the changes in nuclear morphology during topography-induced differentiation, the circularity index, elongation, nucleus area, and volume were measured in hESCs. As significant enhancement on neuronal differentiation was previously observed on 250 nm gratings,(Ankam et al., 2013, 2015) the hESCs were cultured on 250 nm gratings and unpatterned control in neuronal differentiation medium on Day 1, 4, and 7. The temporal analysis of the circularity index (Figure 2B) showed that the undifferentiated cells were more circular (0.82 ± 0.01) as compared to the cells differentiated on nanogratings on Day 4 and 7 (0.70 ± 0.01 and 0.73 ± 0.01, respectively). By Day 4, the hESCs nuclei on unpatterned substrates were more circular (0.76 ± 0.01), but eventually reached similar circularity (0.72 ± 0.01) as compared to cells grown on gratings by Day 7.

The elongation factor is directly proportional to elongation and is inversely correlated with circularity. Elongation changes in the hESCs nuclei on nano-patterned or unpatterned substrates were most significantly observed within the first 24 h of differentiation (Figure 2C). The elongation reached a high value of 0.73 ± 0.02 on the elongation index for cells on gratings, and 0.54 ± 0.02 for cells on unpatterned substrates by Day 1 of differentiation; the increase in elongation of the hESCs nuclei on nano-patterned substrates on Day 1 was significant as compared to Day 0 Cover Slip control (*p* ≤ 0.001, *N* = 4). Furthermore, the elongation of the hESCs nuclei on nano-patterned substrates was significantly higher (0.67 ± 0.08 and 0.76 ± 0.08 on Days 4 and Day 7, respectively) than for the nuclei of cells grown on unpatterned substrates (0.45 ± 0.02 and 0. 51± 0.08 on Day 4 and Day 7, respectively).

When cultured on nano-patterned substrates, the cells nuclei showed the highest alignment (40.71 ± 3.80%) by Day 7 of neuronal differentiation (Figure 2D). On unpatterned substrates, an increase in nucleus and cell area were observed on Day 4 (258.4 ± 14.5 μm^2^ for nucleus area and 1050.4 ± 139.7 μm^2^ for cell area), but were slightly decreased on Day 7 (211.1 ± 10.9 μm^2^ for nucleus area and 864.1 ± 154.7 μm^2^ for cell area, Figures 2E,F). On the nano-patterned substrates, an increase in nucleus and cell area between Day 1 of differentiation (175.0 ± 16.1 μm^2^ for nucleus and 493.2 ± 43.4 μm^2^ for cell area) to Day 4 (225.4 ± 12.0 μm^2^ for nucleus and 918.7 ± 164.9 μm^2^ for cell area) were observed. There were less changes on nucleus and cell area on Day 7 (237.2 ± 30.4 μm^2^ for nucleus area and 949.0 ± 154.3 μm^2^ for cell area). However, the areas of nucleus and cell were smaller (211.1 ± 10.9 μm^2^ for nucleus area and 864.1 ± 154.7 μm^2^ for cell area) for hESCs grown on unpatterned substrates, in comparison to hESCs grown on patterned substrates (237.2 ± 30.4 μm^2^ for nucleus area and 949.0 ± 154.3 μm^2^ for cell area) on Day 7. On the unpatterned substrates, a decrease was observed for the ratio of nuclear to cytoplasmic area (Figure 2G) from Day 1 (0.49 ± 0.03 for cells on unpatterned substrates, 0.42 ± 0.05 for cells on nanogratings) to Day 4 (0.28 ± 0.02 for cells on unpatterned substrates, 0.30 ± 0.04 for cells on nanogratings). The cells grown on the nano-patterned substrates have significantly lower nuclei volumes as compared to those grown on unpatterned substrates on Day 1 and Day 4 (Figure 2H). The normalized nucleus volume increased significantly from Day 1 (1.68 ± 0.22) to Day 4 (2.38 ± 0.08) and then decreased to smaller volumes by Day 7 (0.81 ± 0.03) for cells grown on unpatterned substrates. On the nano-patterned substrates, nucleus reached the highest value of 1.63 ± 0.18 by Day 4 of differentiation and then decreased 1.5 folds (1.09 ± 0.05) by Day 7 of neuronal differentiation.

### Temporal changes in the nucleus morphology and size during neuronal differentiation of hMSCs

**Figure 3:**
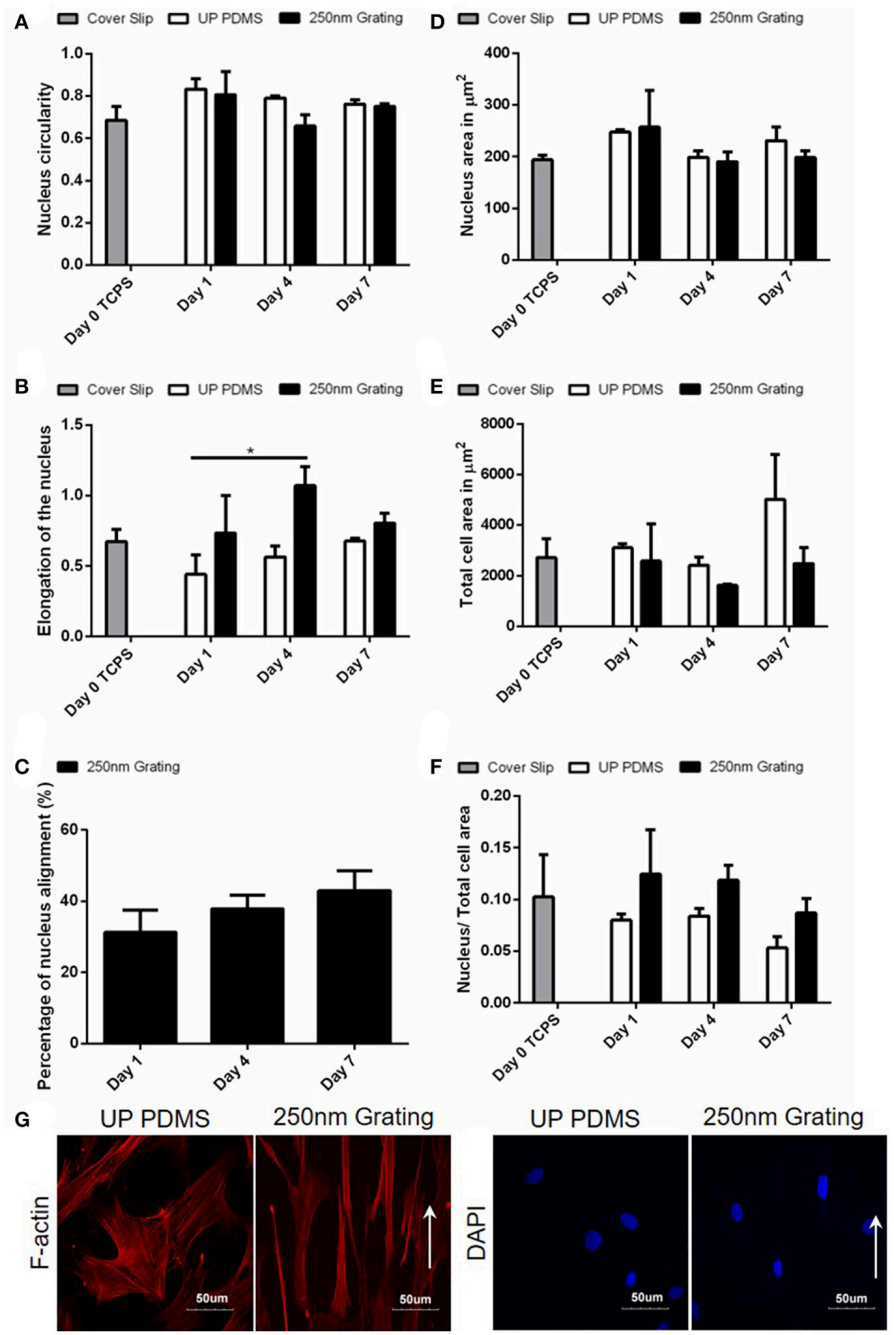
Temporal changes of the **(A)** nucleus circularity (comparison of replica mean values, *N* = 3 for Day 0, 4, 7, and *N* = 2 for Day 1), **(B)** nucleus elongation (comparison of replica mean values, *N* = 3 for Day 0, 4, 7, and *N* = 2 for Day 1), **(C)** nucleus alignment (comparison of experimental replicas, *N* = 2), **(D)** area of nucleus, **(E)** total cell, and **(F)** the ratio of nucleus area to total cell area (comparison of experimental replicas for **(D–F)**, *N* = 4 for Day 0, *N* = 3 for Day 4, 7, and *N* = 2 for Day 1) were measured on hMSCs grown on unpatterned and nano-patterned substrates during differentiation. ^*^Represents statistical significance with *p* ≤ 0.05 against unpatterned PDMS. Data are shown as average ± SEM. Total number of cells analyzed for Day 0: 127, Day 1 unpatterned PDMS: 18, Day 1 250 nm grating: 32, Day 4 unpatterned PDMS: 69, Day 4 250 nm grating: 86, Day 7 unpatterned PDMS: 91, Day 7 250 nm grating: 116. (G) Fluorescence image of hMSCs F-actin cultured on 250 nm gratings PDMS for 7 days. Cellular alignment and elongation were observed on gratings topography. Gratings directions were shown by the white arrows. The elongation Factor *E* = (long axis/short axis) −1.

Human MSCs cultured on TCPS (Day 0) were used as negative controls while cells cultured with retinoic acid on both patterned and unpatterned were treated as positive control for neuronal differentiation (Figures 3A–F). The temporal analysis of the hMSCs nuclei circularity index (Figure 3A) showed that the Day 0 undifferentiated cells were slightly more circular (0.69 ± 0.11) as compared to the cells on nanogratings on Day 4 (0.66 ± 0.09), while the cells on nanogratings on Day 1 and Day 7 have higher circularity (0.81 ± 0.15 and 0.75 ± 0.02, respectively); however, the differences between time points were not found to be statistically significant. This result agrees with what we found in topography screening with MARC chip (Figure 1) where the circularity index between nuclei on unpatterned substrates and on nanogratings topography were not found to be statistically different after 7 days. Consistently, the circularity index of all experimental groups hover around 0.70.

The extent of the equimomental eclipse lengthening of cell nucleus was quantified by the elongation factor, E, which was calculated by the following formula: long axis/short axis −1. It is, in fact, a more sensitive measurement to analyze nuclei elongation changes caused by topographical cues such as nanogratings (Andersson et al., 2003). For an ellipse with short axis: long axis ratio of 1:2, E is equal to 1, while for a perfect circle, E is equal to 0. By using fluorescence images of the cell nucleus, we were able to measure the short and long axes of the cell nuclei. The elongation of hMSCs nuclei reached statistical significance after 4 days in culture (1.07 ± 0.23) as compared to cells nuclei on Day 1 on unpatterned substrates (0.44 ± 0.20) (*p* ≤ 0.05, *N* = 2 for Day 1 group and *N* = 3 for Day 4 group). The elongation of nuclei was, however, reduced from Day 4 (1.07 ± 0.23) to Day 7 (0.81 ± 0.12) on nanogratings topography (Figure 3B).

Similar to the trend observed in hESC culture, on nanogratings topography, hMSC nuclei showed the highest alignment (43.01 ± 7.80%) by Day 7 (Figure 3C). On unpatterned substrates, an increase in nucleus and cell area were observed on Day 7 (230.7 ± 46.4 μm^2^ for nucleus area and 5024.5 ± 3049.0 μm^2^ for cell area). For cells on nanogratings, there was no significant change in nucleus area on Day 4 (190.3 ± 32.0 μm^2^) and on Day 7 (198.5 ± 22.6 μm^2^) as compared to the undifferentiated Day 0 control (194.0 ± 17.6 μm^2^). However, the total cell area on nanogratings topography was reduced on Day 4 (1619.5 ± 91.7 μm^2^) and on Day 7 (2485.4 ± 1078.4 μm^2^) as compared to the Day 0 control (2724.2 ± 1464.2 μm^2^) (Figures 3D,E). The changes in total cell area on nanogratings was better reflected in Figure 3F where the ratio between nuclear to cytoplasmic area was taken. After 7 days of culture, hMSCs were fluorescently stained for F-actin and nuclei. Figure 3G showed cellular alignment of hMSCs on nanogratings substrate which is consistent with the previously reported data (Teo et al., 2013).

### Nanotopography affects early changes in nuclear lamin A/C

Studies in the past have clearly indicated the structural role of nuclear lamins to nuclear envelope (Sullivan et al., 1999). Interestingly, recent studies have shown lamin A/C to be a novel marker for stem cell differentiation, indicating the role of lamin proteins in other cellular functions (Constantinescu et al., 2006; Melcer et al., 2012). However, the expression of lamin A/C during neuronal differentiation of hESCs on unpatterned PDMS and coverslip samples were only weakly detected (Supplementary Figure 8), compared to 250 nm gratings on PDMS. Clear and positive expression occurred only during later stage of differentiation on Day 14 (Supplementary Figure 9) but not on Day 7. While the intensity of lamin A/C expression in hESCs during differentiation was not quantified, the immunofluorescence images show that the lamin A/C expression increased in hESCs as they differentiated.

**Figure 4:**
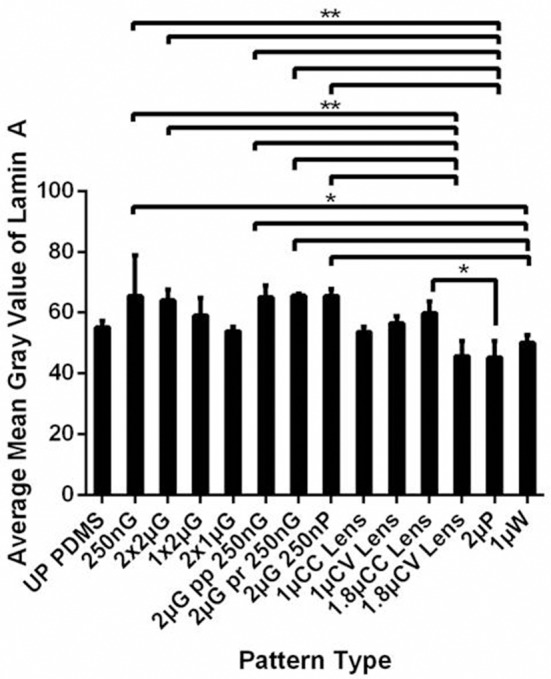
Lamin A/C level was analyzed as the mean gray value of hMSCs cultured on the MARC chip for 7 days. All data shown as average ± SD. Comparison of experimental replicas (*N* = 3) with at least 100 cells were analyzed in each of the experimental replica on each pattern type. ^*^Represents statistical significance with *p* ≤ 0.05 and ^**^p ≤ 0.01. Refer to Table 1 for abbreviations.

Human MSCs are at a more advanced stage in the differentiation and therefore it will be interesting to characterize the expression of lamin A/C in hMSCs during the topography induced differentiation. From the MARC chip analysis, hMSCs on the different topography showed a different level of lamin A/C expression with the lowest expression mostly occurred on isotropic pattern such as 1.8 μm convex lenses, 2 μm pillars (*p* ≤ 0.01 against expression on 250 nm gratings), and 1 μm wells (*p* ≤ 0.05 against expression on 250 nm gratings). It is interesting to note that lamin A/C expression on 250 nm gratings is considerably higher than unpatterned PDMS control and its average is the highest among the different topographies (Figure 4).

**Figure 5:**
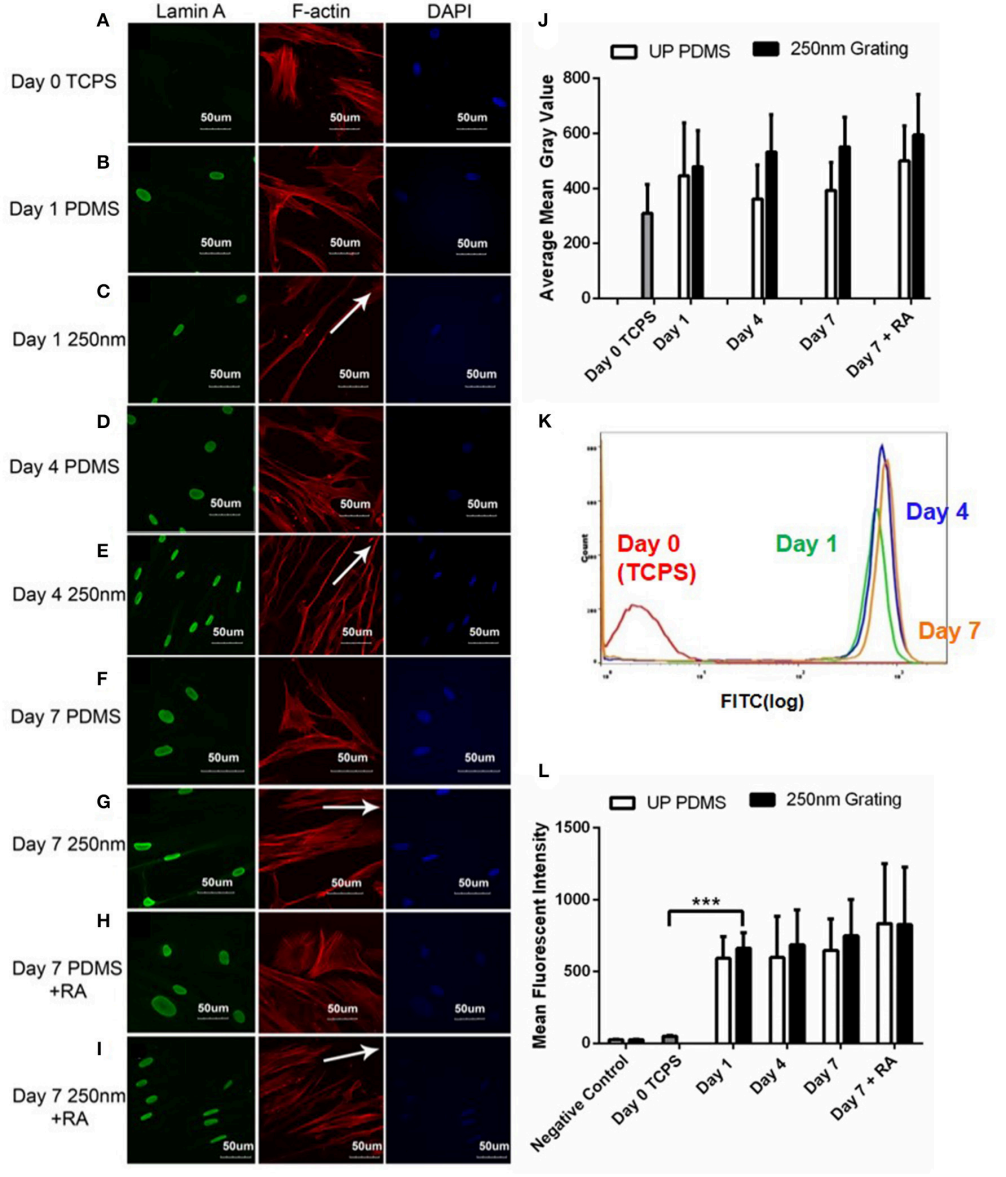
Confocal microscopy images of hMSCs immunofluorescently labeled for lamin A/C (green), F-actin (red) and nuclei (DAPI, blue) on 250 nm PDMS and unpatterned PDMS substrates on Day 1 **(B,C)**, 4 **(D,E)**, and 7 **(F,G)**. The hMSCs on Day 0 cultured on tissue culture polystyrene (TCPS) were used as negative controls **(A)** while retinoic acid (RA) was added to induce neuronal differentiation for positive controls **(H,I)**. All scale bars represent 50 μm while arrows represent grating axis. **(J)** ImageJ intensity quantification of lamin A/C in hMSCs over 7 days. **(K)** Representative population histogram overlay for hMSCs on 250 nm gratings and the corresponding lamin A/C expression. Graphs for Day 0 (red), Day 1 (green), Day 4 (blue) and Day 7 (orange) are shown in different respective colors. **(L)** Flow cytometry analysis of lamin A/C expression in hMSCs cultured on 250 nm PDMS and unpatterned PDMS substrates for different time periods. Positive controls were supplemented with retinoic acid (RA). The points plotted are the mean fluorescent intensity (M.F.I) ± S.D. Comparison of experimental replicas (*N* = 3) with more than 100 cells were analyzed in each of the experimental replica. All data shown as average ± SD. ^***^Represents statistical significance with *p* ≤ 0.001. All results were statistically different vs. Day 0 TCPS with at least *p* < 0.05 except for negative control.

The expression of lamin A/C was observed as a thick band around the nuclear envelope (Figures 5A–I). Confocal images showed temporal changes (Day 0 to Day 7) in the qualitative expression of lamin A/C during neuronal differentiation of hMSCs. Lowest levels of lamin A/C were observed in undifferentiated cells on Day 0 while highest lamin A/C expression was observed in cells differentiated on nanogratings for 7 days (Figures 5B–G). Interestingly, retinoic acid (RA) treatment did not affect the expression of lamin A/C. cells cultured on nanogratings with or without retinoic acid did not have significantly different lamin A/C expression. Expression of lamin A/C and histone methylation in hMSCs grown on nanogratings increased over the 7-day differentiation period. Also, hMSCs grown on nanogratings expressed higher level of lamin A/C compared to corresponding cells grown on unpatterned substrates.

Quantitative and temporal changes in lamin A/C expression were analyzed using flow cytometry. Again, no significant increase in lamin A/C expression was observed with RA induction (Figure 5L). However, the results indicated a dramatic increase in the number of cells positively stained for lamin A/C and significant increase in intensity just after 1 day of hMSC culture on nanogratings. This reiterates that topography induced nuclear matrix remodeling and epigenetic changes leading to differentiation occur quite early.

### Topographical modulation on H3K9 methylation on hMSCs using MARC chip

**Figure 6:**
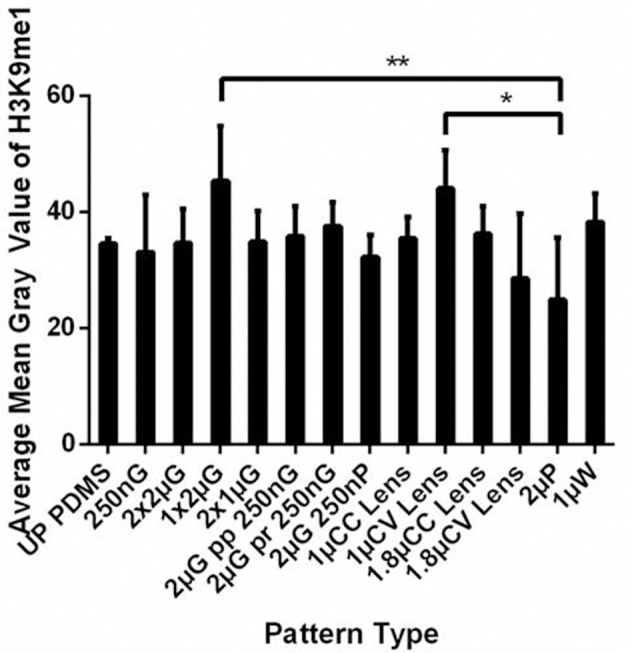
The histone 3 lysine 9 monomethylation (H3K9me1) level was analyzed as the mean gray value of hMSCs cultured on the MARC chip for 7 days. All data shown as average ± SD. Comparison of experimental replicas (N = 4) with at least 100 cells were analyzed in each of the experimental replica on each pattern type. ^*^ *p* represents statistical significance with *p* ≤ 0.05, ^**^ *p* ≤ 0.01. Refer to Table 1 for abbreviations.

The significant changes in topography induced hMSC nuclei elongation encouraged us to study the effect of topography on histone methylation, specifically histone 3 lysine 9 monomethylation (H3K9me1). The percentage of hMSCs with H3K9me1 positive and the mean gray value of the H3K9me1 expression of the hMSCs cultured on the MARC chip were measured on Day 7 after differentiation (Figure 6). Interestingly, different expression level was observed in the hMSCs differentiated on various patterns, for example, with increased H3K9me1 expression on 1 μm gratings, 1 μm convex lenses and 1 μm wells, and the lowest level on 2 μm pillars, compared to unpatterned controls. However, trend on changes according the feature size (micro vs nano) or geometry (isotropic vs anisotropic) was not observed. We speculate that the difference in the H3K9 methylation level could be changed among various differentiation time-points.

### H3K9me3 and H3K9ac decreases during neuronal differentiation of hESCs

**Figure 7:**
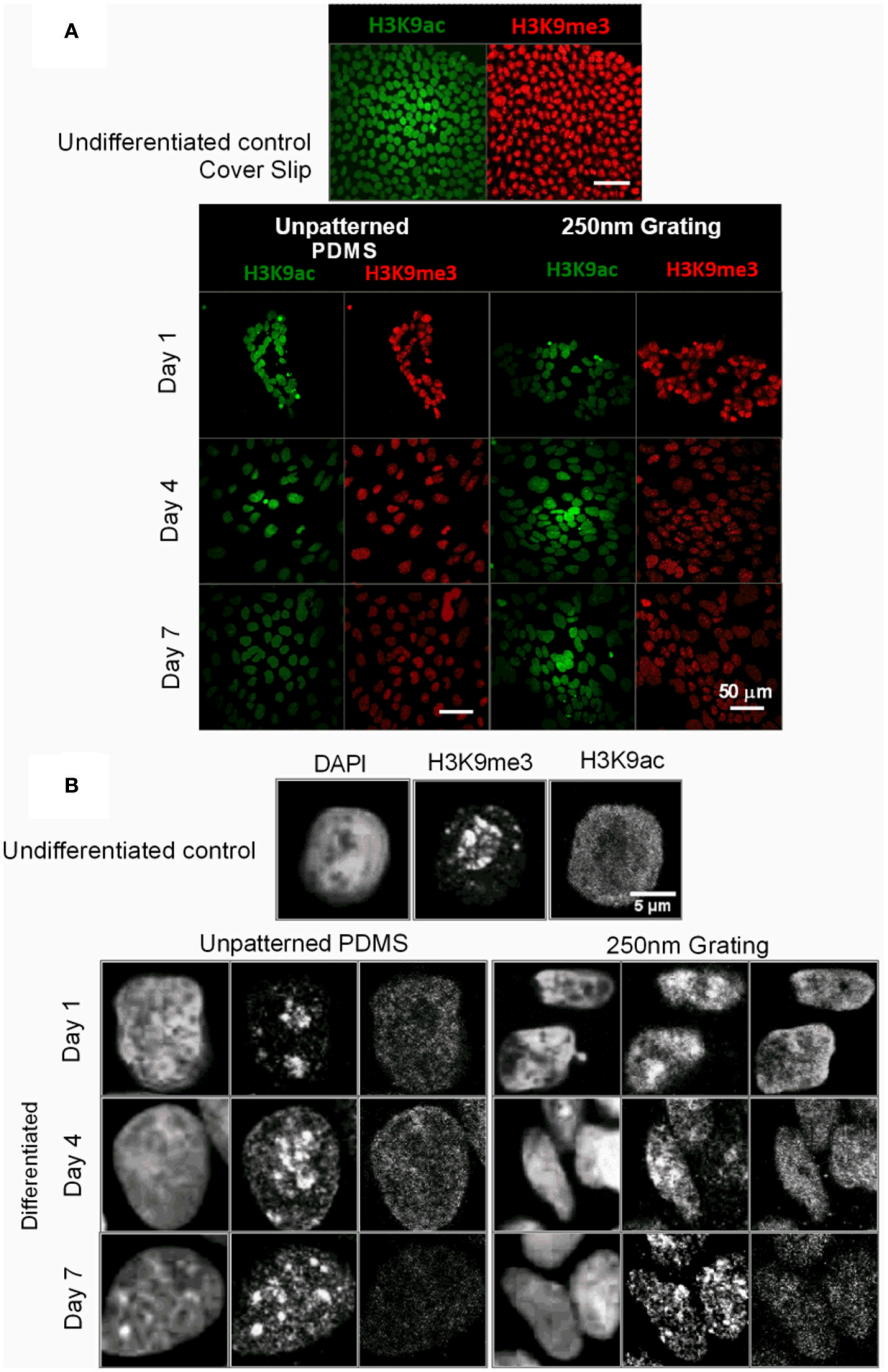
**(A)** The undifferentiated hESCs and hESCs at different time point of differentiation were immunostained for histone methylation (H3K9me3) in red and acetylation marks (H3K9ac) in green. The scale bar represents 50 μm. **(B)** Higher magnification images showed the changes in organization and the punctate expression of H3K9me3 in the nuclei of hESCs during the early phase of neuronal differentiation.

Histone modifications are correlated to specific changes in biological function (Peterson and Laniel, 2004). Human ESCs grown on unpatterned and nano-patterned substrates were immunostained for histone 3 Lysine 9 trimethylation (H3K9me3) and histone 3 Lysine 9 acetylation (H3K9ac) temporally. Higher expression of H3K9me3 and H3K9ac was observed in undifferentiated hESCs. Both, acetylation and methylation marks decreased during neuronal differentiation of hESC on both patterned and unpatterned substrates (Figure 7A). The histone markers H3K9me3 were expressed as punctate and H3K9ac expression was diffuse in the nuclei (Figure 7B). Semi-quantitative immunofluorescence intensity of H3K9ac and H3K9me3 were quantified and included as Supplementary Figure 10.

### Nanotopographical cues modulates H3K9me1 expression in hMSCs

The H3K9me1 is enriched at the transcriptional start site of active genes (Barski et al., 2007) and it is a key mark in the establishment of functional heterochromatin (Rivera et al., 2014). Furthermore, the modifications of H3K9me1 and H3K9me2 have been shown to be involved in neurodevelopment (Ebbers et al., 2016) and neuronal differentiation (Hirano and Namihira, 2016; Laurent et al., 2016).

**Figure 8:**
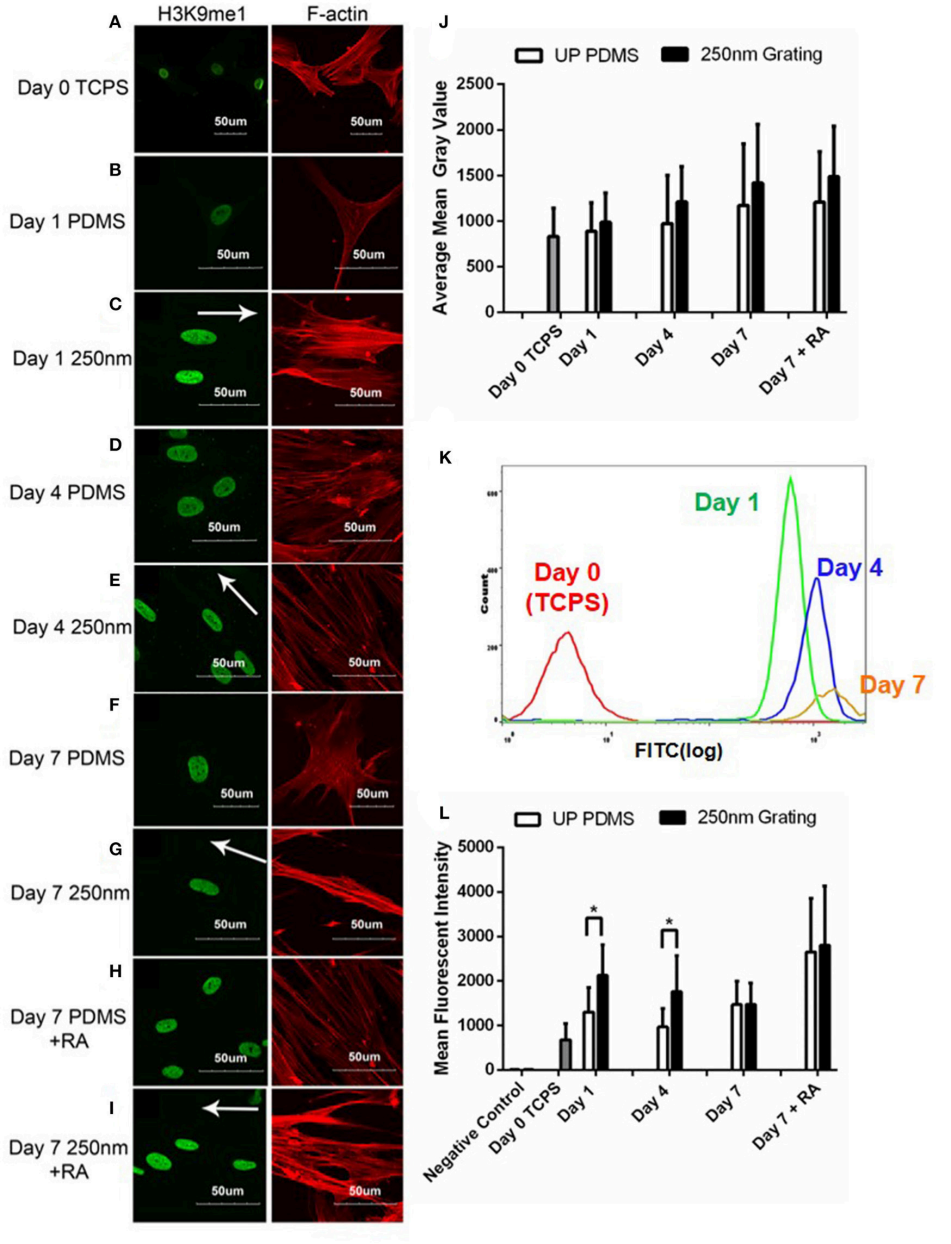
Confocal microscopy images of hMSCs immunofluorescently labeled for H3K9me1 (green) and F-actin (red) on unpatterned PDMS and 250 nm PDMS gratings on Day 1 **(B,C)**, 4 **(D,E)**, and 7 **(F,G)**. HMSCs on Day 0 cultured on tissue culture polystyrene (TCPS) were used as negative controls **(A)**, while retinoic acid (RA) was added to induce neuronal differentiation for positive controls **(H,I)**. All scale bars represent 50 μm while arrows represent grating axis. **(J)** ImageJ intensity quantification of H3K9me1 in hMSCs over 7 days. **(K)** Representative population histogram overlay for hMSCs on 250 nm nanogratings and the corresponding H3K9me1 expression. Graphs for Day 0 (red), Day 1 (green), Day 4 (blue), and Day 7 (orange) are shown in different respective colors. **(L)** Flow cytometry analysis of H3K9me1 expression in hMSCs cultured on 250 nm PDMS and unpatterned PDMS substrates for different time periods. Positive controls were supplemented with retinoic acid (RA). The points plotted are the mean fluorescent intensity (M.F.I) ± S.D. Comparison of experimental replicas (*N* = 3) with at least 100 cells were analyzed in each of the experimental replica. ^*^represents statistical significance with *p* ≤ 0.05. All 250 nm nanogratings were statistically different vs. Day 0 TCPS with *p* ≤ 0.05.

Confocal images were used for temporal qualitative analysis of H3K9me1 expression in hMSCs grown on patterned and unpatterned substrates. Cells cultured on TCPS (Figure 8A) were used as negative controls while cells cultured with retinoic acid on both patterned and unpatterned were treated as positive control (Figures 8H,I). Overall, temporal increase in the expression of H3K9me1 was observed (Figure 8B–G). The fluorescence images were quantified using ImageJ (Figure 8J).

Intensity of H3K9me1 expression was lowest in cells grown on TCPS and increased temporally during the 7-day culture. Notably, nanogratings induced the highest expression of H3K9me1 when compared to cells cultured on unpatterned PDMS substrates for the same period of time. Contrasting to lamin A/C expression, retinoic acid increased the expression of H3K9me1 in hMSCs cultured on both unpatterned and patterned substrates after 7 days of culture. These results suggest that nanotopography might play a synergistic role with biochemical cues (RA) in increasing expression of H3K9me1 and in turn hMSC differentiation.

In order to quantitatively determine the difference in H3K9me1 expression levels at different time points, cells seeded on unpatterned and patterned substrates with and without retinoic acid were analyzed for H3K9me1 expression using flow cytometry. The mean fluorescent intensity of H3K9me1 expression in hMSC were plotted (Figure 8L). These results corresponded with the semi-quantitative analysis of confocal images for immunofluorescence intensity levels where an increasing level of H3K9me1 expression was observed between Day 0 and Day 7 in hMSCs grown on nanogratings. Overall, 250 nm gratings induced the highest expression of H3K9me1 in hMSCs when compared to cells cultured on TCPS substrates at all corresponding time points. Cells cultured on nanogratings showed a higher expression of H3K9me1 when compared to their corresponding unpatterned substrates on Day 1 and Day 4. However, on Day 7, no changes in expression levels of H3K9me1 were observed in cells cultured on either nanogratings or unpatterned substrates, with or without RA induction.

Figure 8K indicates the heterogeneity of H3K9me1 expression in hMSCs on nanotopography analyzed at various time points. This might indicate the varying levels of differentiated state on the nanogratings. These results contrast the lamin A/C histograms which have a narrow width compared to H3K9me1 expression levels, indicating the heterogeneity in methylation. Again, the cells showed a dramatic increase in number of positively stained and intensity of H3K9me1 expression within the first 24 h, suggesting that topography induced differentiation starts early on.

## Discussion

Studies on nanotopography-induced differentiation on pluripotent stem cells (Abagnale et al., 2013; Ankam et al., 2013, 2015; Chan et al., 2013a; Pan et al., 2013; Song et al., 2016; Chen et al., 2018) and adult stem cells (Yim et al., 2007; Qi et al., 2013; Teo et al., 2013, 2014; Newman et al., 2016) from our group and others, have demonstrated the physical cue, the substrate topography, can enhance neuronal differentiation of stem cells. We observed a significant influence of micro- and nano-topography on the neuronal differentiation of stem cells and the synergistic response to the neuronal differentiation when the nanotopography would be combined with biochemical cues. To understand the mechanism of topography-induced neuronal differentiation, we have previously demonstrated that the cytoskeletal contractile and focal adhesion signaling play an essential roles in the topography-sensing and mechanotransduction in the topography-induced neuronal differentiation (Teo et al., 2013; Ankam et al., 2015). The aim of this study is to understand how stem cells nuclear regulation is involved in the nanotopography-induced differentiation. Hence, in this study, the nuclear changes in both hESCs and hMSCs on nanotopography were studied using experimental design with the minimal biochemical signals for the differentiation to elucidate the effects mainly contributed by the topography. We hypothesized that the nucleus morphology and H3K9 methylation will be changed, and the expression level of lamin A/C will increase when stem cells are cultured on the 250 nm PDMS compared to the control flat PDMS surface.

High nuclear to cytoplasmic ratio (0.5) is characteristic of undifferentiated hESCs (Thomson et al., 1998). The nucleus is coupled to actin and other intermediate filaments that transduce signals from the extracellular matrix or substrate to the nucleus (Maniotis et al., 1997). The increase in cytoplasmic area and the decrease in the nuclear to cytoplasmic ratio observed in hESCs grown on unpatterned and nanogratings on Day 4 suggests that the cells are preparing for differentiation by increasing in size. Similar decrease in the nuclear to cytoplasmic ratio was observed in hMSCs grown on nanogratings on Day 7. In the case of hESCs, this change corresponded to the highest increase in nuclear volume on Day 4 of differentiation in hESCs grown on nanogratings and unpatterned substrate. During embryonic stem cell differentiation, global rates of transcription change by varying the volume of nucleus, increase or decrease in volume depending on the differentiation fate, while the transcription site density remains constant (Faro-Trindade and Cook, 2006). When looking at the dynamics of changes in nucleus volume, the cells grown on unpatterned substrates behave very differently when compared to the cells grown on nanogratings. This leads to speculation that hESC differentiated on nanogratings may adopt a different pathway when compared to cells cultured on unpatterned substrates in reaching a neuronal fate.

Using the MARC chip, we observed changes in the circularity of stem cell nuclei induced by various topographical patterns. Previously, it has been shown that ESC nuclei were highly deformable and the lack of nuclear matrix protein lamin A/C contribute to the softer nuclear structure of pluripotent stem cells (Pajerowski et al., 2007); a larger range of difference in nucleus circularity was observed in the hESC nuclei. The hESCs cultured on anisotropic patterns had nuclei with lower circularity. Meanwhile, the circularity of nuclei in hMSCs was not significantly affected by the underlying topographical substrate, with exceptions observed on convex lenses and pillars where the circularity of the hMSC nuclei was significantly reduced. However, when measuring the elongation of the nuclei, the nuclei of the hESCs and hMSCs cultured on nanogratings were significantly more elongated compared to the stem cells cultured on the unpatterned controls.

Significant elongation of the actin cytoskeleton and nucleus was observed in the direction of nanogratings as compared to the cells that were randomly aligned on unpatterned substrates. It could be speculated that the elongation of stem cells (both hMSCs and hESCs) nuclei when grown on topographical substrates could be caused by the physical confinement within the channel or by the mechanical coupling of the cytoskeleton and nucleus. Since the nanogratings used in this study were 250 nm wide, it is more likely that changes in the nucleus shape could be attributed to cytoskeletal changes (Teo et al., 2013; Ankam et al., 2015). The actin filaments are bi-directionally connected to the ECM through integrins and on the other end to the nuclear membrane through the SUN/KASH complex (Zhang et al., 2001; Crisp et al., 2006). While the detailed mechanism of mechanotransduction remains a topic of active research and investigation, the relationship and coordination between cytoskeletal arrangement and stress and the nuclear shape have been studied in differentiated cells such as cardiac myocytes (Lee et al., 2015) and endothelial cell (Versaevel et al., 2012). A recent study on the cytoskeletal regulation on nuclear state shows that actomyosin stress fibers and intermediate filaments modulate the mechanical properties of the nucleus and chromatin condensation, while the myosin intracellular pulling forces regulate nuclear volume and morphology (Keeling et al., 2017).

Furthermore, the structural organization of the nucleus could be physically controlled by nanotopography as evidenced by LINC, a specialized structure that links nucleus and cytoskeleton (Fey et al., 1984; Crisp et al., 2006). Studies have shown that mechanical forces transduced through LINC might also be involved in regulating critical DNA enzymes or factors (Maniotis et al., 1997). The mechanical decoupling of integrins and nuclei was further demonstrated by the disruption of intermediate filaments. Studies have also shown that forces applied to apical integrins are transmitted to basal focal adhesions and nucleus, indicating the physical connection between the ECM and nucleus (Hu et al., 2003, 2005). In a study conducted by Dalby et al., fibroblasts grown on topographies showed spatial alteration of chromosomes (Dalby et al., 2007a). Changes in nuclear shape and corresponding changes in gene expression have not only been observed by our group (Yim et al., 2007) but by several other research groups as well (Dalby et al., 2003, 2007a). Lamin network mechanically interfaces the actin cytoskeleton with chromatin (Dalby et al., 2007a) and our work suggests that these substrate topography related changes occur at an early time-point. This communication between the ECM and nucleus allows chromatin to be regulated. Cellular forces from topography could be transduced to unravel DNA motifs or cryptic binding sites in mechanosensitive plasma proteins, directing stem cell function and behavior.

Lamin A/C has been shown to be a marker for differentiated ESCs (Constantinescu et al., 2006; Melcer et al., 2012), and modulate differentiation of ESCs. We observed a very weak expression of lamin A/C during neuronal differentiation of hESCs on PDMS and coverslip samples. A stronger lamin A/C expression was detected during later stage of differentiation with retinoic acid on Day 14. The low lamin A/C expression in undifferentiated hESCs was not expected as other studies demonstrated that the lamin A/C levels are very low in undifferentiated cells (Thomson et al., 1998; Constantinescu et al., 2006; Sehgal et al., 2013). Lamin A/C expression was also observed in the positive controls with 10 μM of retinoic acid.

The semi-quantitative temporal analysis using immunofluorescence staining and the quantitative flow cytometric analysis of fluorescence intensity results confirmed that fluorescence intensity of lamin A/C increased from Day 0 to Day 7 in hMSCs. An increase in the expression of lamin A/C was observed from Day 0 to Day 7 for both the 250 nm gratings and control substrate. The results also suggested that 250 nm gratings were able to induce larger changes in the expression of lamin A/C as compared to the unpatterned controls at the same timepoint, thereby suggesting a greater influence by nanotopography-induced differentiation of hMSCs. As expected, the Day 7 samples with retinoic acid added as an additional biochemical differentiation cue showed the higher expression of lamin A/C, confirming its role as a positive control in the experiment.

As shown previously, lamin A/C expression can be modulated by retinoic acid, through its binding to RARE (Retinoic acid responsive element) present on the lamin A promoter (Okumura et al., 2004). Another study has shown the mechanosensitive regulation of lamin A expression in the presence of retinoic acid on soft vs. stiff gels (Swift et al., 2013). These help us in understanding the variation in the levels of lamin A/C expression seen in the adult stem cell (hMSC) vs. the embryonic stem cell state (hESC). Furthermore, Andres et al. have reviewed the interaction of lamin A and lamin B receptor with nuclear envelope and how they regulate the unique architecture of heterochromatin by using rod photoreceptor cells (Andrés and González, 2009). Hence, we investigated the interaction between lamin A/C and heterochromatin by looking into the expression pattern of histones.

A significant increase in the mean fluorescence intensity of lamin A/C and H3K9me1 expression in hMSCs on nanogratings was observed on Day 1. Interestingly, this observation also aligned with the significant changes in nuclear elongation of the hESCs on nanogratings on Day 1. Although the observed effect could be due to differences in the mechanical properties between the PDMS substrate and the culture dishes (on Day 0), we speculate that the difference observed in the nuclear morphology and lamin A expression on Day 1 would not be due to the different mechanical properties between PDMS and TCPS. Previous studies have shown differences in cell behavior on softer vs. stiffer substrates within the physiological relevant range of rigidity (Weng and Fu, 2011; Palchesko et al., 2012; Yip et al., 2013). However, the PDMS used in our study was much stiffer than the stiff hydrogel used in these studies, while Yip et al. (2013) and Ghibaudo et al. (2008) also found that beyond physiological rigidity (100 kPa), cells traction force and focal adhesion area remained constant and resulted in indifferent cell behavior regardless of substrate stiffness beyond this point. Furthermore, if the changes in lamin A expression would be caused by the difference in substitute rigidity, as TCPS is much stiffer than PDMS, the lamin A expression should be decreased. Hadden et al. showed that lamin A expression strongly increased in intensity between 2 and 18 kPa in a dose-dependent manner, but the increase was slower above 18 kPa (Hadden et al., 2017). In addition, to verify that the difference would not be due to the mechanical properties between the PDMS and TCPS, we quantified the average nucleus area and elongation of hESCs and hMSCs in conventional maintenance culture condition on both TCPS and PDMS. No significant difference was observed in the nuclear morphology between the stem cells cultured on the two substrates (Supplementary Figure 5). Images of nuclei on both the substrates were also shown in Supplementary Figure 5 for qualitative comparison. Meanwhile, nuclear lamin A has been demonstrated to be an important mechano-sensor for matrix stiffness and the regulation of stem cell differentiation and lineage commitment (Cho et al., 2017; Ivanovska et al., 2017). Another study showed micro-groove topography induced lamin B reorganization and changes in lamin A/C gene expression in immortalized BJ fibroblasts (McNamara et al., 2012). Lamin A/C could therefore be one possible downstream target in transducing both topographical and stiffness cues from the substrate.

Topography induced differentiation of stem cells causes both morphological and temporal changes in histone markers. Histone modifications are important for epigenetic regulation to stem cell differentiation (Dai and Rasmussen, 2007; Xie et al., 2013; Qiao et al., 2015). As the pluripotent hESCs and the adult multipotent MSCs are at different stages of differentiation, the temporal changes in the early events of nanotopography-induced differentiation were also different between hESCs and hMSCs. In hESCs, the expression of H3K9me3 and H3K9ac were strong in the undifferentiated population and decreased once the differentiation initiated. As the hESC differentiation progressed, the H3K9me3 distribution and expression level increased. H3K9me3 had a diffuse expression on Day 1 and became better organized as punctate with higher expression in the hESC nuclei on Day 7 on nanogratings (Figure 7). DNA methylation is highly active in undifferentiated ES cells while expression of key developmental genes is accompanied with demethylation (Lagarkova et al., 2006; Yeo et al., 2007). H3K9 methylation is associated with transcriptional repression and H3K9 acetylation is correlated with increased histone deposition and gene expression (Peterson and Laniel, 2004). The qualitatively higher expression observed in this study on undifferentiated hESCs might indicate the transcriptionally repressed state.

On the contrary, adult MSCs exhibited a different temporal expression of histone methylation and lamin A/C expression, as MSCs are more committed in their differentiation potential compared to the pluripotent hESCs. A semi-quantitative temporal analysis using immunofluorescence staining and quantitative flow cytometry analysis in hMSCs showed an increase in the expression H3K9me1. A significantly higher expression of H3K9me1 was observed for hMSCs on nanogratings topography compared to the corresponding unpatterned substrates on Day 1 and Day 4. However, there was no difference in H3K9me1 expression between hMSCs on nanogratings topography and on unpatterned substrates after 7 days, with or without RA induction. This observation agrees with the level of H3K9me1 expression of hMSCs on the different topographies in the MARC chip analysis (Figure 6) which shows no statistical significant difference against the unpatterned controls.

Apart from topography, other studies have also shown the role of stiffness in modulating hMSC differentiation (Engler et al., 2006). Also, it is evident from our study that RA supplementation acts synergistically with topography in increasing H3K9me1 expression in hMSCs.

Significant changes in H3K9me1 expression levels that were observed between Day 0 and Day 1 were consistent with previous studies that demonstrated the fate specification of hMSCs depending on the early exposure to mechanical cues (Fu et al., 2010). This was further supported by another study that indicated FAK activity in hMSCs was crucial during the first 24 h of exposure to topography in inducing differentiation (Teo et al., 2013). Collectively, epigenetic and cytoskeletal changes can take place as early as 24 h when exposed to mechanical cues like topography or stiffness.

These studies combined with our own findings allow us to better understand the role of 250 nanogratings in inducing neuronal differentiation of hMSCs and hESCs. Further research to decouple the effects of substrate stiffness and topographical cues needs to be carried out. More in-depth studies and quantitative characterization on the changes in histone expression will be needed to understand the epigenetic regulation involved in the nanotopography-induced differentiation. Our previous studies showed that nanotopography induced changes in integrin clustering and focal adhesion signaling, thereby transducing the mechanical forces from the substrate to the actin cytoskeleton. This change in cytoskeletal contractility could play a significant role in influencing gene expression due to its physical connection with the nucleus. This study has significantly contributed to understanding the coupling between the actin cytoskeleton and the nucleus, specifically its interaction with lamins and histones, in pluripotent and adult stem cells. This information could provide more efficient ways to regulate or direct stem cell fate for therapeutic applications.

## Author contributions

EY, SA, and BT were responsible for conception and design of the study. SA, BT, SH, and CL performed data collection. SA, BT, GP, and EY were responsible for data analysis and interpretation. EY, SA, BT, and GP were mainly responsible for writing the manuscript. All authors approve this final version of the article.

### Conflict of interest statement

The authors declare that the research was conducted in the absence of any commercial or financial relationships that could be construed as a potential conflict of interest.
