# Supplementary material for: Temporal Changes in Nucleus Morphology, Lamin A/C and Histone Methylation During Nanotopography-Induced Neuronal Differentiation of Stem Cells
Source: Front Bioeng Biotechnol. 2018 May 31;6:69. doi: 10.3389/fbioe.2018.00069 (PMC5990852; doi:10.3389/fbioe.2018.00069)
Supplement: Supplementary file 1 [file Data_Sheet_1.PDF]

Supplementary Material

Temporal changes in nucleus morphology, lamin A/C and histone methylation during nanotopography-induced neuronal differentiation of stem cells

Soneela Ankam, Benjamin Kim Kiat Teo, Grace Pohan, Shawn Wei Loong Ho, Choon Kiat Lim, Evelyn King Fai Yim*

*** Correspondence:** Corresponding Author: eyim@uwaterloo.ca

1 Supplementary Data

1.1 Materials and methods of immunofluorescence analysis of pluripotency and neuronal markers of hESCs

The human embryonic stem cells (hESCs) samples were fixed in 4% paraformaldehyde (Sigma) and permeabilized in 0.2% Triton X. The samples were then blocked in 10% goat serum and 1% bovine serum albumin (BSA) for 1 hour before overnight incubation with primary antibodies at 4ºC. The antibodies for pluripotency and neuronal markers for the hESC characterization, and the dilutions used in this study can be referred from Table S.5. The samples were stained for F-actin using phalloidin (Invitrogen) tagged to Alexa Fluor 633 and counterstained with DAPI (Invitrogen) at 2µg/ml.

The samples were mounted using Prolong Gold anti-fade mounting reagent (Invitrogen) and imaged using Leica epi-fluorescence microscope or Leica TCS SP5 confocal microscope.

Table S.1. List of antibodies used in this study is listed.

Antibody	Dilution	Source/Company
mouse anti-SSEA4	1:200	Santa Cruz
mouse anti-Tra-1-60	1:200	Santa Cruz
rabbit anti-Oct4	1:500	Abcam
rabbit anti-nanog	1:200	Santa Cruz
rabbit anti-Tuj1	1:750	Sigma
mouse anti-MAP2	1:500	Abcam

rabbit anti-pMLC	1:200	Cell signalling

1.2 Materials and methods of extended neuronal differentiation of hESCs for lamin A/C examination

Human embryonic stem cell line H1 (WiCell Institute, Madison, http://www.wicell.org) was routinely cultured in a feeder free system using hESC-qualified matrigel (BD Biosciences) and mTeSR1 medium (Stem Cell Technologies). The medium was changed once daily. Human ESCs were passaged enzymatically with dispase (1 mg/ml) after manual removal of differentiated colonies, using an inverted microscope (Nikon TS100) and flame bent Pasteur pipette. The cells were passaged at a ratio of 1:4 – 1:6.

The hESCs were directly seeded on poly-L-ornithine (PLO) and laminin-coated cover slip or patterned and unpatterned PDMS. It was estimated that about 10 - 15 clumps ($2x10^4$ – $2.5x10^4$ cells) were needed to be seeded per cm^2 of PDMS substrate. The hESCs were cultured in N2B27 medium (DMEM/F12 (Bio-Rev Pte. Ltd): Neurobasal medium (Invitrogen) in the ratio 1:1 supplemented with 1X N2 and B27 supplements (Invitrogen)) for 14 days before fixation and immunofluorescence staining. To enhance neuronal differentiation, 10μM of retinoic acid (RA) was added to the medium of the hESCs cultured on cover-slip as a control. The samples were Samples were immunofluorescently stained for lamin A/C with mouse anti-lamin A/C (1:100) and either rabbit-anti- neuron-specific class III β-tubulin (Tuj1) (1:750) or rabbit-anti- phospho-Myosin Light Chain (pMLCK). Samples were then incubated with the fluorescent Alexa Fluor 546 goat anti-mouse, and Alexa Fluor 488 goat anti-rabbit secondary antibodies (1:500, Molecular Probes) at room temperature for 1 hour. The samples were stained for F-actin using phalloidin (Invitrogen) tagged to Alexa Fluor 633 and counterstained with DAPI (Invitrogen) at 2μg/ml.

2 Supplementary Figures

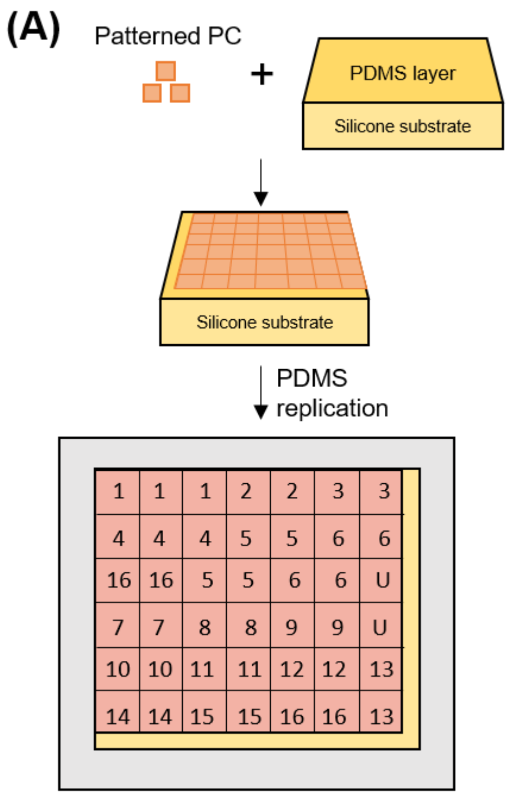

Topo-graphy number	MARC chip topography	Abbreviations
1	250 nm lines, 250 nm space, 250 nm depth	250nG
2	250 nm lines, 250 nm space, 110 nm depth	-
3	460 nm lines, 70 nm space, ~40 nm height	-
4	2 µm lines, 2 µm space, 2 µm height	2x2µG
5	1 µm lines, 2 µm space, 120 nm height	1x2µG
6	2 µm lines, 1 µm space, 80 nm height	2x1µG
7	2 µm line ⊥ 250 nm line	2µG pp 250nG
8	2 µm line // 250 nm line	2µG pr 250nG
9	2 µm lines 250 nm pillar	2µG 250nP
10	1 µm pitch 0.3 µm sag microlens concave	1µCC Lens
11	1 µm pitch 0.3 µm sag microlens convex	1µCV Lens
12	1.8 µm diameter, 2 µm pitch, 0.7 µm sag microlens concave	1.8µCC Lens
13	1.8 µm diameter, 2 µm pitch, 0.7 µm sag microlens convex	1.8µCV Lens
14	2 µm pillars, 12 µm pitch, 2 µm height	2µP
15	1 µm holes, 6.5 µm pitch, 1 µm depth	1µW
16	250 nm pillar, 400 nm pitch, 250 nm height	-
U	Unpatterned control	UP PDMS

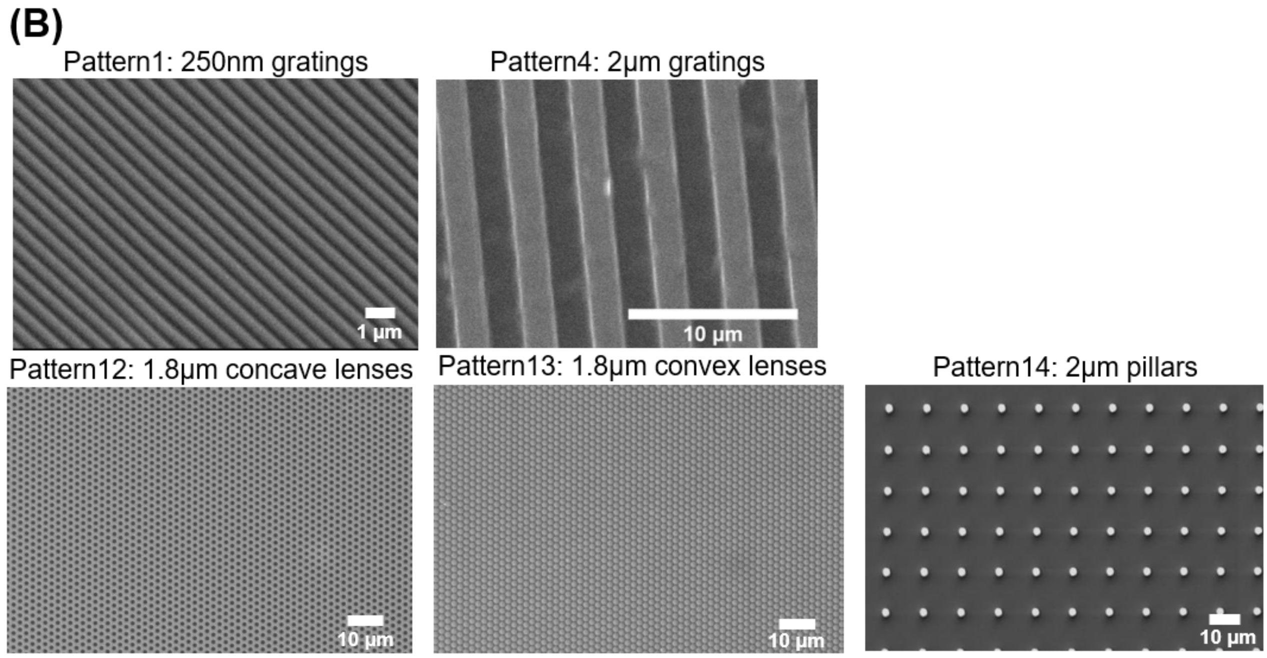

Supplementary Figure 1. (A) 16-pattern Multi-architecture (MARC) Chip map with topography description and abbreviations. (B) Scanning electron microscopy (SEM) images of selected patterns on the MARC Chip.

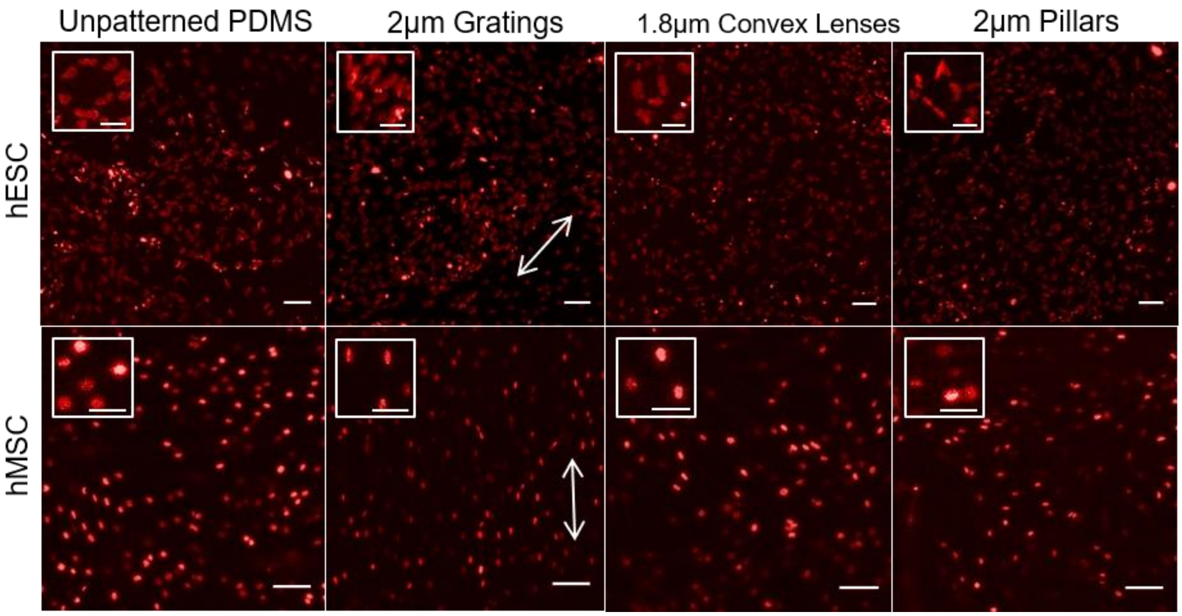

Supplementary Figure 2. Nuclei shape of hESC and hMSC on 2μm gratings, 1.8 μm convex lenses and 2μm pillar topography. Double arrows indicate the gratings direction and inserts show the zoom-in images of nuclei. Scale bar of main images: 50 μm, scale bar of inserts: 25 μm.

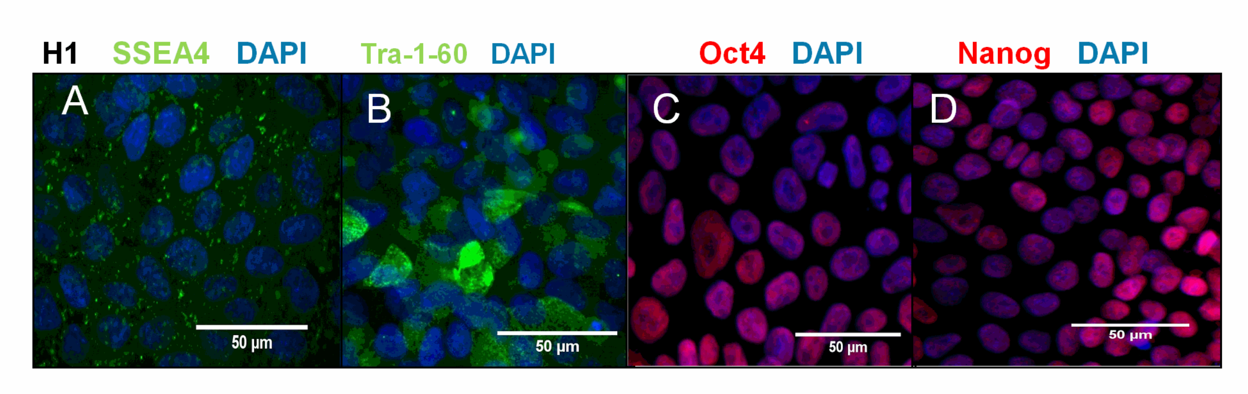

Supplementary Figure 3. Expression of pluripotency markers in undifferentiated H1 human embryonic stem cells (hESCs).

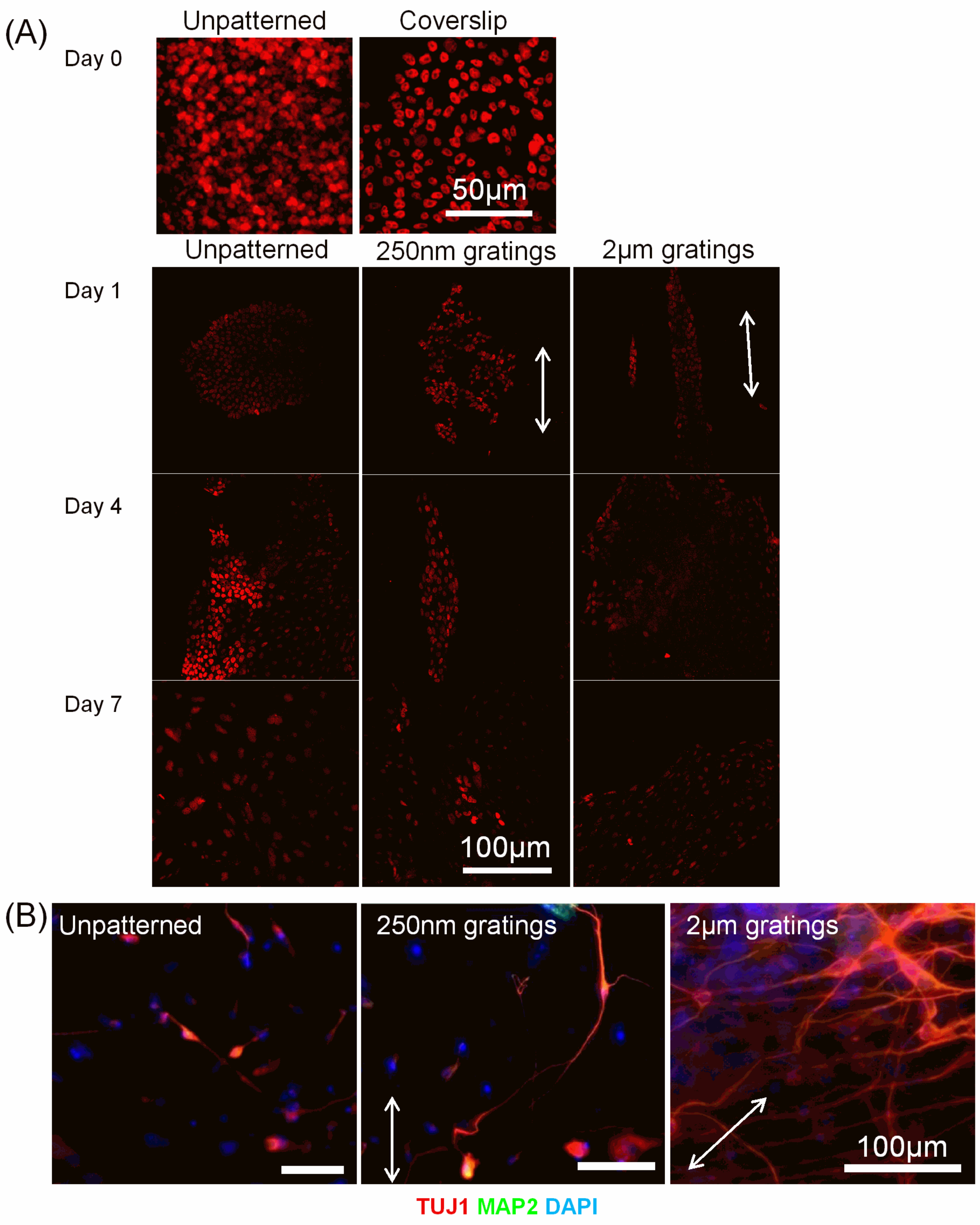

Supplementary Figure 4. Characterization of hESC's (A) pluripotency marker (Nanog) and (B) neuronal markers (neuron-specific class III β-tubulin, Tuj1; microtubule-associated protein 2, MAP2) expression. Gratings axes are shown by the white arrows. Scale bar of Supplementary Figure 4B: 100 μm.

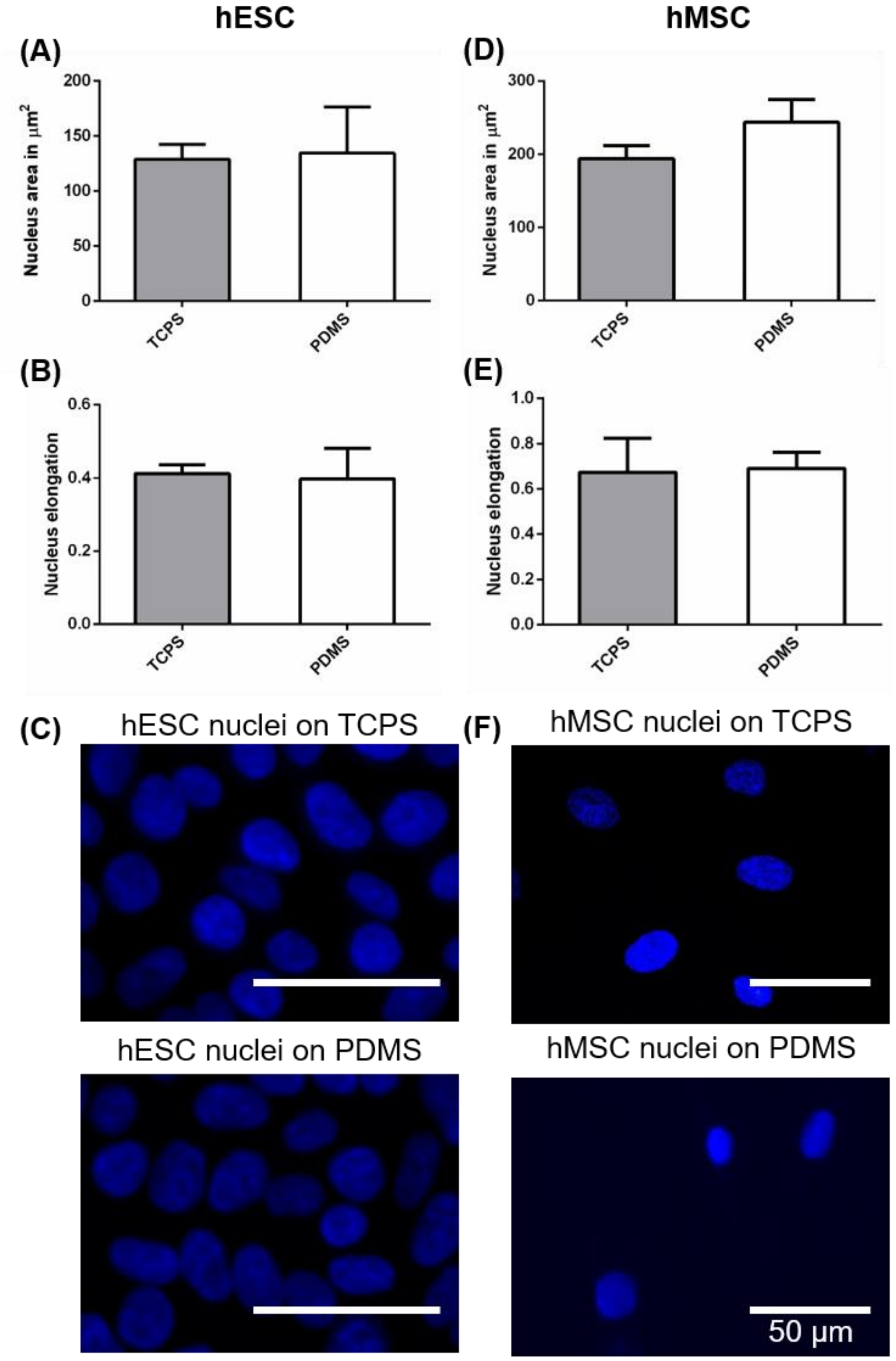

Supplementary Figure 5. Comparison between hESCs nuclei (A) area (comparison of replica mean values, N=3 and N=2 for TCPS and PDMS, respectively, with at least 50 nuclei analyzed in each replica) and (B) elongation (comparison of replica mean values, N=4 and N=2 for TCPS and PDMS, respectively, with at least 50 nuclei analyzed in each replica) and hMSCs nuclei (D) area (comparison of replica mean values, N=2 and N=2 for TCPS and PDMS, respectively, with at least 50 nuclei analyzed in each replica) and (E) elongation (comparison of replica mean values, N=2 and

N=2 for TCPS and PDMS, respectively, with at least 50 nuclei analyzed in each replica) on TCPS and PDMS substrate with unpatterned topography. Data are shown as average ± SD. No statistical significance was found between TCPS and PDMS groups. Immunofluorescence images of (C) hESCs and (F) hMSCs nuclei stained with DAPI on both substrates were shown. Scale bar: 50 µm.

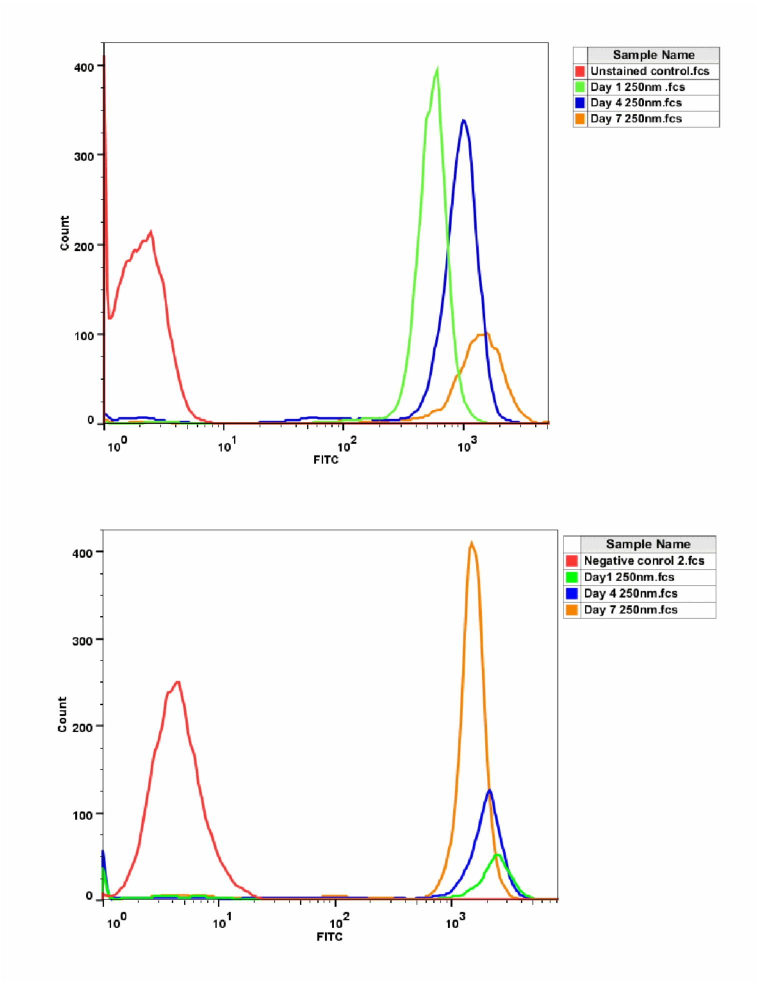

Supplementary Figure 6. Representative flow cytometry analysis of histone H3 mono methyl K9 (H3K9me1) expression in human mesenchymal stem cells (hMSCs) at different time points.

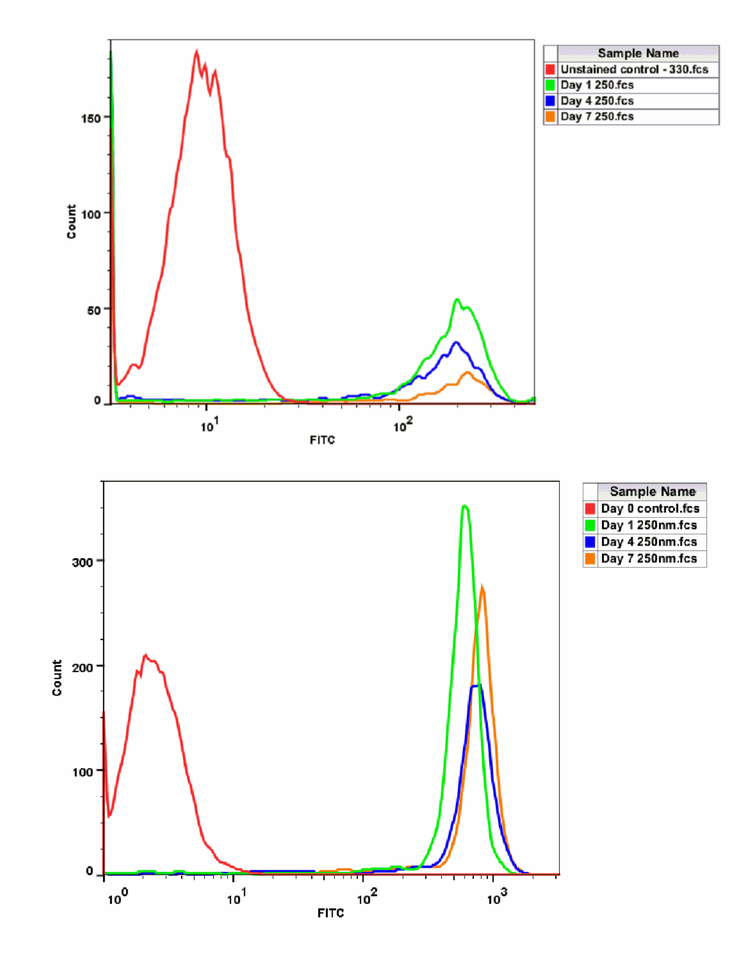

Supplementary Figure 7. Representative flow cytometry analysis of lamin A/C in hMSCs at different time points.

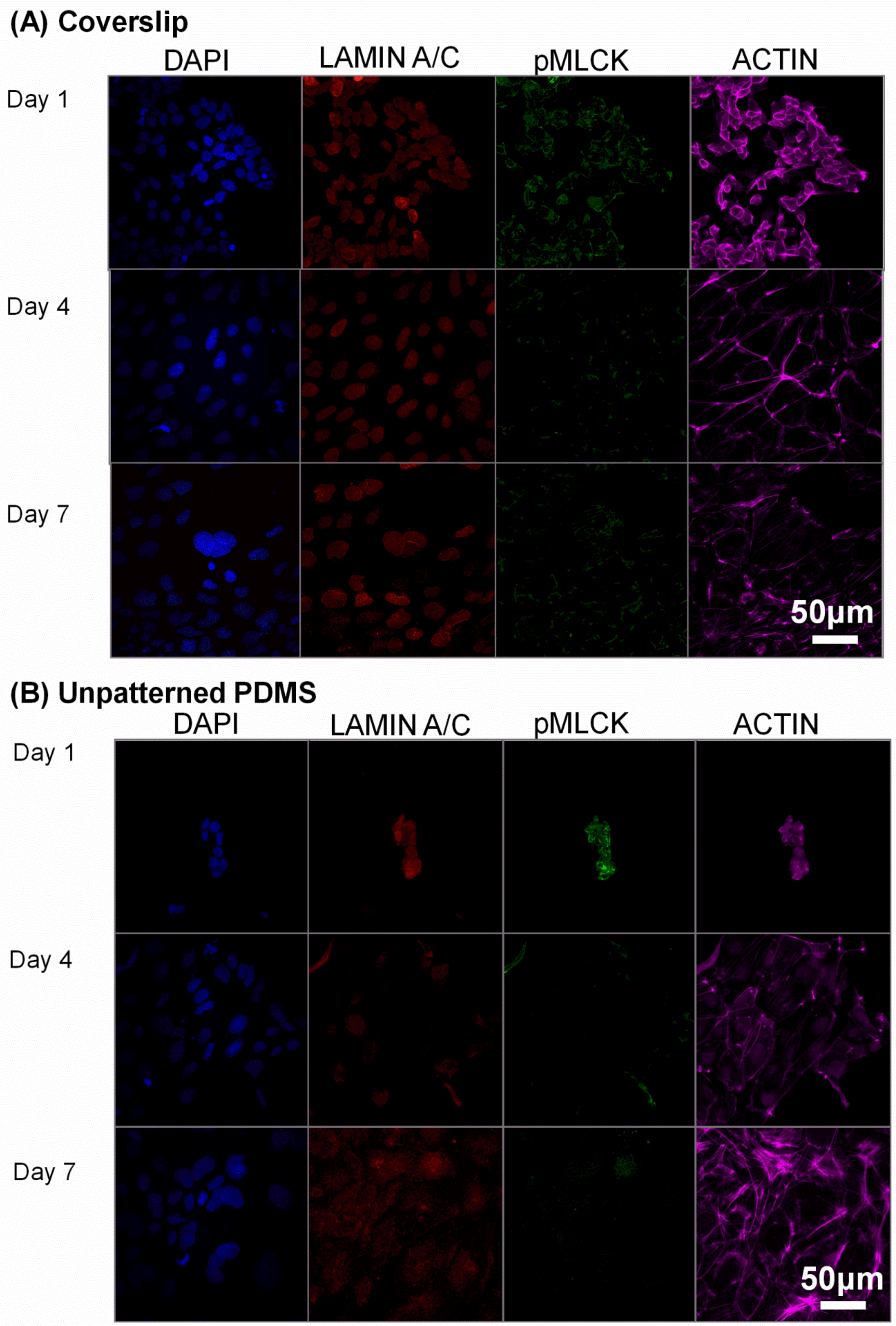

(A) Coverslip
DAPI
LAMIN A/C
pMLCK
ACTIN
Day 1
Day 4
Day 7
50µm
(B) Unpatterned PDMS
DAPI
LAMIN A/C
pMLCK
ACTIN
Day 1
Day 4
Day 7
50µm

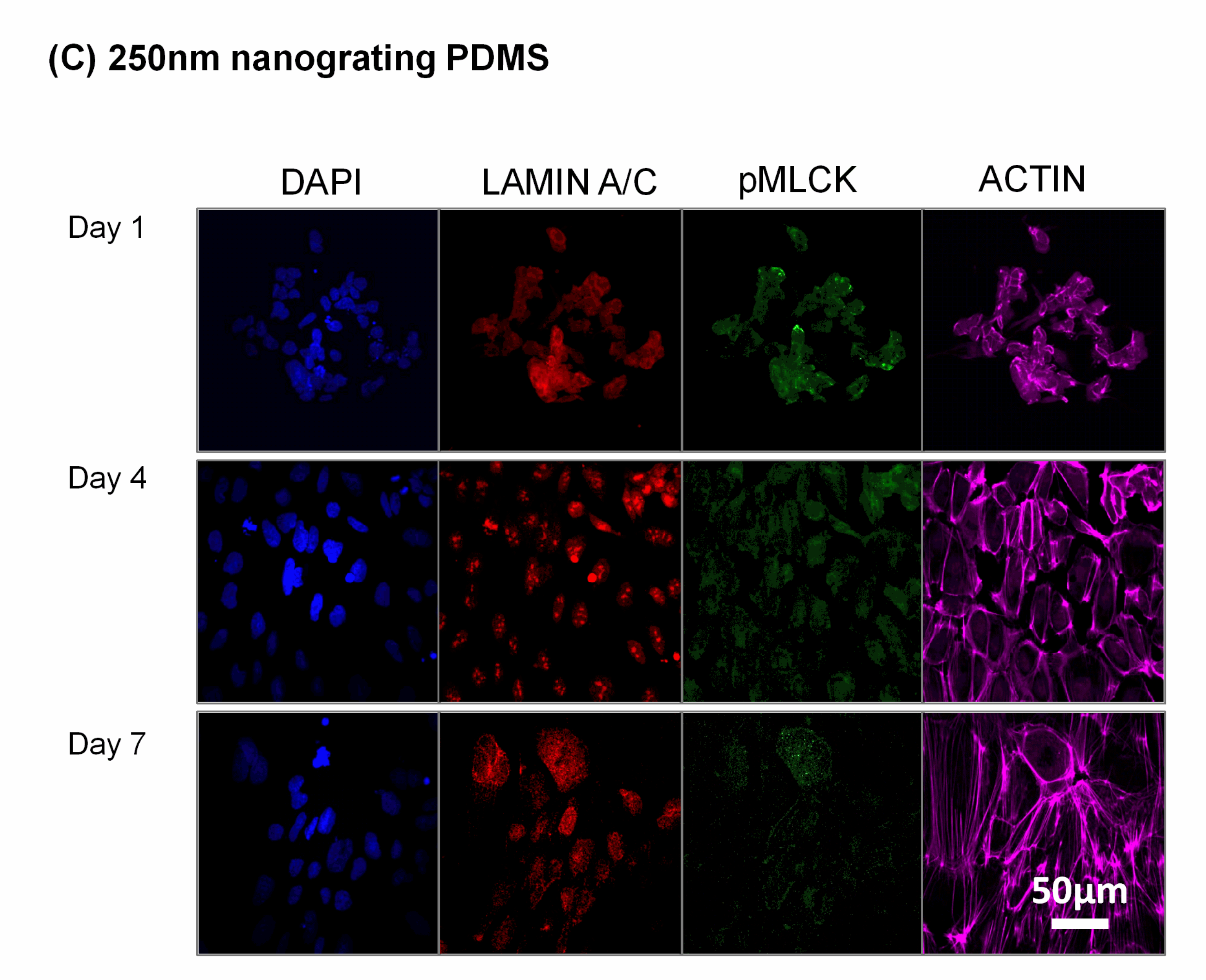

Supplementary Figure 8. Lamin A/C expression (red) of hESCs on (A) Cover Slip, (B) Unpatterned PDMS, and (C) 250nm gratings PDMS substrates, with co-staining of phospho-Myosin Light Chain (pMLCK, green), F-actin (magenta), and DAPI (blue)

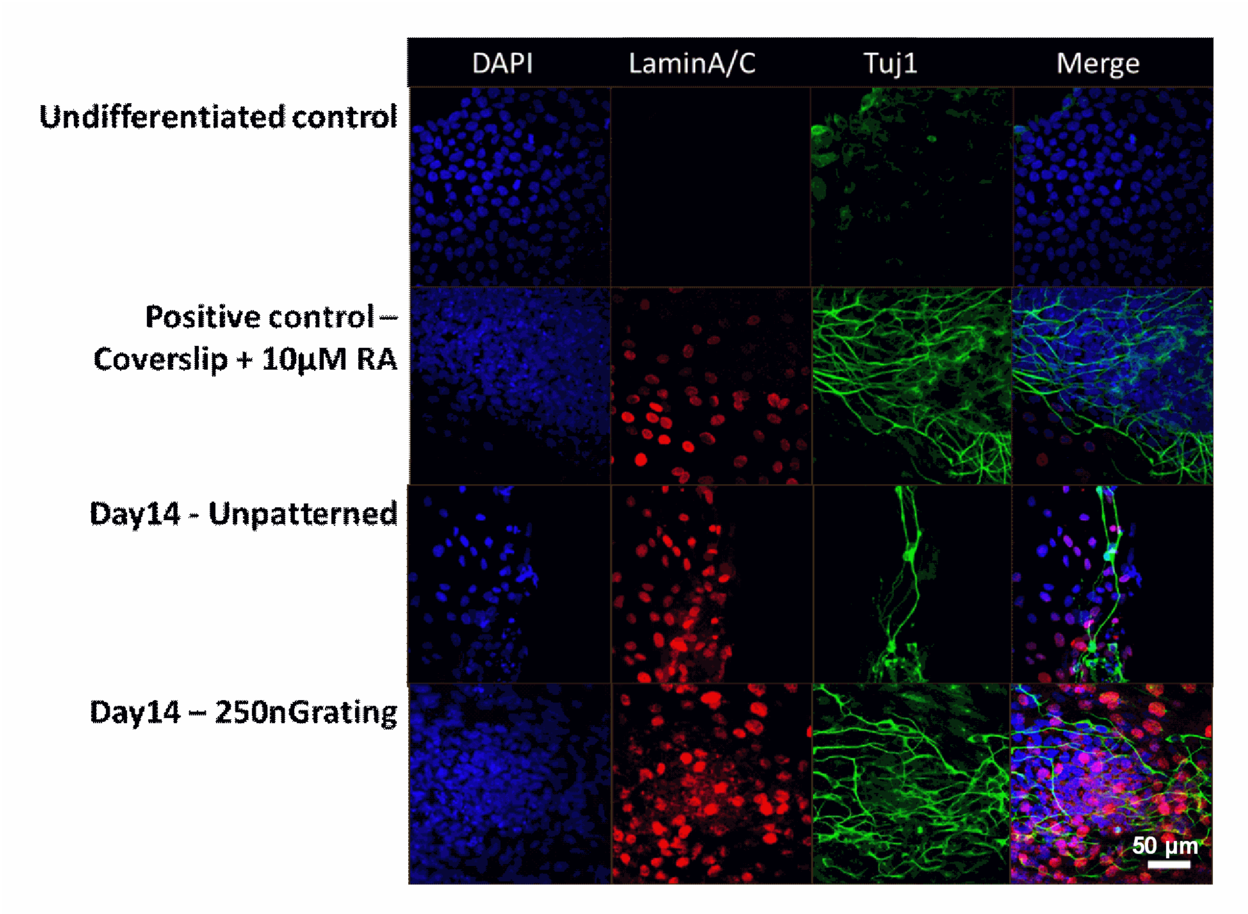

Supplementary Figure 9. Lamin A/C expression in hESCs which were differentiated in neuronal differentiation media for 14 days on unpatterned and 250nm grating PDMS. Human ESCs grown on coverslips with retinoic acid (RA) is shown as the positive control, while undifferentiated hESCs are shown as the negative control. The hESCs are immunostained for lamin A/C in red, Tuj1 in green and DAPI in blue. Scale bar: 50 μm.

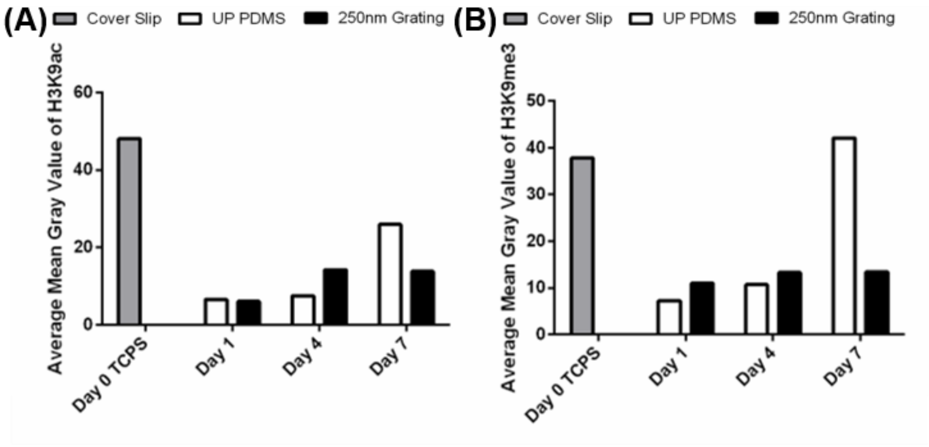

Supplementary Figure 10. Semi-quantitative ImageJ intensity quantification of (A) H3K9ac and (B) H3K9me3 in hESCs over 7 days. Data are shown as average. N=1 with at least 37 nuclei were analyzed per experimental group.
